# Phylogenetics and Taxonomy of the Fungal Vascular Wilt Pathogen *Verticillium*, with the Descriptions of Five New Species

**DOI:** 10.1371/journal.pone.0028341

**Published:** 2011-12-07

**Authors:** Patrik Inderbitzin, Richard M. Bostock, R. Michael Davis, Toshiyuki Usami, Harold W. Platt, Krishna V. Subbarao

**Affiliations:** 1 Department of Plant Pathology, University of California Davis, Davis, California, United States of America; 2 Graduate School of Horticulture, Chiba University, Matsudo, Chiba, Japan; 3 Agriculture and Agri-Food Canada, Charlottetown Research Centre, Charlottetown, Prince Edward Island, Canada; University of Missouri-Kansas City, United States of America

## Abstract

Knowledge of pathogen biology and genetic diversity is a cornerstone of effective disease management, and accurate identification of the pathogen is a foundation of pathogen biology. Species names provide an ideal framework for storage and retrieval of relevant information, a system that is contingent on a clear understanding of species boundaries and consistent species identification. *Verticillium*, a genus of ascomycete fungi, contains important plant pathogens whose species boundaries have been ill defined. Using phylogenetic analyses, morphological investigations and comparisons to herbarium material and the literature, we established a taxonomic framework for *Verticillium* comprising ten species, five of which are new to science. We used a collection of 74 isolates representing much of the diversity of *Verticillium*, and phylogenetic analyses based on the ribosomal internal transcribed spacer region (ITS), partial sequences of the protein coding genes *actin* (*ACT*), *elongation factor 1-alpha* (*EF*), *glyceraldehyde-3-phosphate dehydrogenase* (*GPD*) and *tryptophan synthase* (*TS*). Combined analyses of the *ACT*, *EF*, *GPD* and *TS* datasets recognized two major groups within *Verticillium*, Clade Flavexudans and Clade Flavnonexudans, reflecting the respective production and absence of yellow hyphal pigments. Clade Flavexudans comprised *V. albo-atrum* and *V. tricorpus* as well as the new species *V. zaregamsianum*, *V. isaacii* and *V. klebahnii*, of which the latter two were morphologically indistinguishable from *V. tricorpus* but may differ in pathogenicity. Clade Flavnonexudans comprised *V. nubilum*, *V. dahliae* and *V. longisporum*, as well as the two new species *V. alfalfae* and *V. nonalfalfae*, which resembled the distantly related *V. albo-atrum* in morphology. Apart from the diploid hybrid *V. longisporum*, each of the ten species corresponded to a single clade in the phylogenetic tree comprising just one ex-type strain, thereby establishing a direct link to a name tied to a herbarium specimen. A morphology-based key is provided for identification to species or species groups.

## Introduction

The genus *Verticillium* comprises a small group of plant-pathogenic fungi that cause billions of dollars of damage annually to a variety of agricultural crops in many parts of the world [1]. *Verticillium* species are soil-borne and cause Verticillium wilt, a plant disease that affects the vasculature of many different hosts [1], and can cause significant crop losses [2].

Control of Verticillium wilt is difficult and costly [3], [4]. In the absence of a suitable plant host, *Verticillium* species can remain dormant in the soil for years by means of small, melanized resting structures that are extremely durable, and will only germinate in the proximity of a suitable host [5].

*Verticillium* has a long taxonomic history. The first species of *Verticillium* was first found in 1816 [6], and approximately 190 species have since been described [7]. The species share the characteristic *Verticillium* conidiophore that is comprised of spore-forming cells that are narrowly flask-shaped, and are assembled into whorls (verticils) and attached along a main axis. The advent of molecular systematics confirmed that *Verticillium* was composed of several distantly related and ecologically diverse groups which were subsequently removed from *Verticillium* [8], [9] and placed in other genera. These include *Lecanicillium*, containing insect and fungus pathogens [10], [11], [12], *Pochonia* and *Haptocillium* comprising nematode parasites [13], [14], and *Gibellulopsis* and *Musicillium* containing plant pathogens [15]. The reduced genus *Verticillium*, also referred to as *Verticillium* sensu stricto, thus consisted of only five species of plant associates and plant pathogens [15], and was retypified with *V. dahliae* [16]. *Verticillium* is placed in the family Plectosphaerellaceae [15] that is closely related to *Colletotrichum* in the Glomerellaceae [17], another important group of plant pathogens. Both Plectosphaerellaceae and Glomerellaceae are families of uncertain phylogenetic position in the Hypocreomycetidae, a subclass within the fungal phylum Ascomycota [17], [18]. *Gibellulopsis* and *Musicillium* are also part of the Plectosphaerellaceae [15], whereas *Lecanicillium*, *Pochonia* and *Haptocillium* are placed in different families in the order Hypocreales of the Hypocreomycetidae [19]. *Verticillium* species reproduce only asexually, no sexual state is known [20].

Besides playing an important role in the biology of *Verticillium* species, resting structures are also taxonomically important. Resting structures were first recognized in *V. albo-atrum* as brown, pigmented hyphae described as ‘Dauermycelien’ [21], a term translated to ‘resting mycelium’ by Isaac [22]. Other types of melanized resting structures in *Verticillium* are chlamydospores that consist of short chains of brown, rounded cells, whereas microsclerotia are rounded, brown cells that occur in clusters. Resting mycelium, chlamydospores and microsclerotia are collectively referred to as resting bodies or resting structures [23]. Resting structures were traditionally used as the primary characteristic to distinguish *Verticillium* species. *Verticillium albo-atrum* was defined based on the presence of resting mycelium, *V. nubilum* formed chlamydospores, *V. dahliae* and *V. longisporum* produced microsclerotia, and *V. tricorpus* formed resting mycelium, chlamydospores and microsclerotia simultaneously [22], [23], [24]. Supporting earlier studies that cast doubt on the usefulness of resting structures as a taxonomic character [25], [26], phylogenetic analyses suggested that resting structure morphology may not be a suitable character to identify species. In a recent phylogenetic tree [15], instead of clustering into separate, well-defined groups, four out of five *Verticillium* species overlapped and only one species was confined to a separate group in the tree.

The goal of this study was to create a solid taxonomic framework for *Verticillium*, and to determine whether resting structure morphology is a suitable character for species delimitation in *Verticillium*. The scheme that we developed attaches names to all major, species-level phylogenetic groups in *Verticillium*, and provides a means for their identification. The new taxonomic system allows for a more reliable and consistent identification of species, and will lead to a significant improvement of our knowledge of *Verticillium* biology. Potential practical applications are many, and may include more efficient and effective disease management strategies such as pathogen exclusion and successful quarantine.

Our approach was as follows. We first assembled a diverse collection of *Verticillium* strains to cover much of the known *Verticillium* diversity. We then studied evolutionary relationships and species boundaries using multigene phylogenetic analyses and morphological investigations. Finally, we determined the correct names for the species recovered by comparison to ex-type strains, herbarium material and the literature, and described new species for groups where no names were available.

## Results

### DNA sequence data

In order to investigate the phylogenetic relationships between *Verticillium* species, we generated DNA sequence data for 64 isolates, 317 DNA sequences were submitted to GenBank (Accessions ITS: JN187963–JN188023; *ACT*: JN188088–JN188151; *EF*: JN188216–JN188279; *GPD*: JN188152–JN188215; *TS*: JN188024–JN188087). An attempt to obtain DNA sequence data from the *V. dahliae* type specimen failed, as the DNA extract generated from a small part of the *Dahlia* sp. stem containing *V. dahliae* microsclerotia did not yield any PCR products (data not shown).

### Single-locus analyses

To investigate whether the five single-locus datasets (ITS, *ACT*, *GPD*, *EF*, *TS*) contained similar phylogenetic information, we first analyzed each dataset individually using parsimony. For each single-locus analysis, we included only one representative of each allele. See Table 1 for descriptive statistics of the single-locus analyses. An ITS alignment, and an alignment of the combined *ACT*, *GPD*, *EF* and *TS* datasets, were submitted to TreeBase (http://purl.org/phylo/treebase/phylows/study/TB2:S11756).

**Table 1:** Statistics of the ITS, *ACT*, *EF*, *GPD* and *TS* single-locus datasets, the combined four-locus dataset and their respective most parsimonious trees.

Locus	Haplotypes	Taxa	Characters	Variable characters	Parsimony informative characters	MPTs: number/steps	CI/RI^b^	Clades >70% support
ITS	15	74	514	74 (14%)^a^	62 (12%)^a^	1/94	0.904/0.943	10
*ACT*	17	77	638	283 (44%)	230 (36%)	9/427	0.855/0.925	20
*EF*	19	77	614	338 (55%)	234 (38%)	12/599	0.825/0.896	20
*GPD*	23	77	781	252 (32%)	209 (27%)	2/430	0.802/0.917	27
*TS*	26	77	625	298 (48%)	236 (38%)	396/565	0.772/0.911	25
*ACT*, *EF*, *GPD*, *TS*	32	77	2658	1171 (44%)	996 (27%)	48/2041	0.805/0.944	35

a Percentages refer to the proportions of variable and parsimony informative characters in each dataset.

b CI: consistency index; RI: retention index.

We did not detect any significant conflict between the most parsimonious trees from the five single-locus datasets on a 70% bootstrap support level (Figures S1, S2, S3, S4, S5), with the following exceptions. *Verticillium nubilum* was sister group to the clade of *V. isaacii*, *V. klebahnii*, *V. tricorpus* and *V. zaregamsianum* in the *GPD* tree with 89% bootstrap support (Figure S4), but in the *EF* and *TS* trees, *V. nubilum* was sister group to the clade of *V. alfalfae*, *V. dahliae*, *V. longisporum* and *V. nonalfalfae* with 97% support in both trees (Figures S3, S5). In the majority of trees, *V. zaregamsianum* was sister group to the *V. isaacii*, *V. klebahnii* and *V. tricorpus* clade with 100% support whereas in the *EF* tree (Figure S3), *V. zaregamsianum* was equally distantly related to all other *Verticillium* species. Also, the *EF* tree differed from the *TS* tree in the position of Species A1, an unknown ancestral species of the diploid hybrid *V. longisporum* [27]. Species A1 was a sister group to the clade of Species D1, another unknown ancestral species of *V. longisporum* [27] and *V. dahliae* in the *TS* tree with 76% support (Figure S5), whereas in the *EF* tree, Species A1 was sister to the monophyletic group of *V. alfalfae*, *V. nonalfalfae*, *V. dahliae* and Species D1 that were supported by 99% of the bootstrap replicates (Figure S3). In the remaining single-locus trees, the position of Species A1 was not fully resolved (Figures S1, S2, S4).

### Combined analyses

**Figure 1:**
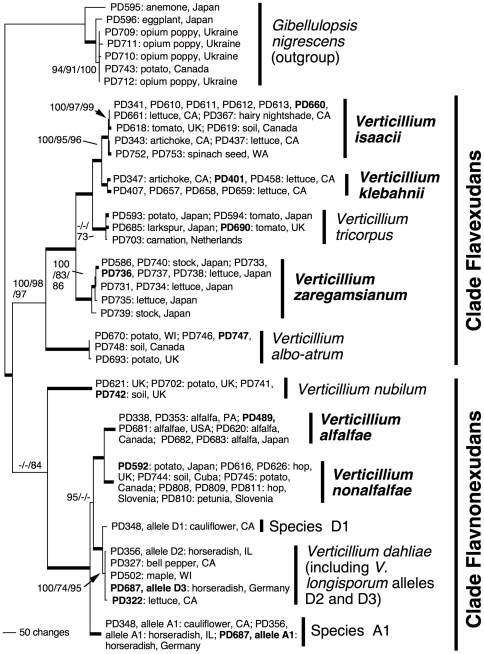
Phylogenetic relationship of the ten *Verticillium* species based on the combined *ACT*, *EF*, *GPD* and *TS* dataset of 2658 characters and 77 taxa, with *Gibellulopsis nigrescens* as outgroup. The Bayesian consensus tree is shown. Isolates are represented by their unique PD identifiers followed by host and geographic origin, PD identifiers in bold represent ex-type strains. Species are marked by vertical bars followed by species names, species in bold were described in this study. The two main clades recovered are indicated on the right. Numbers by the branches are Bayesian, likelihood and parsimony support values above 70 in that order, branches in bold had maximal support in all analyses. For the diploid hybrid *V. longisporum*, allele designations are also given following PD identifiers. Each isolate of *V. longisporum* has two alleles that are present in two different clades in the tree, in hypothetical Species A1, and either in hypothetical Species D1 or in *V. dahliae*, reflecting the hybrid origin of this species [27]. Groupings not visible in the tree but still receiving support include the clade of strains PD710 and PD743 with 71% bootstrap support, the clade of strains PD356, PD327 and PD502, with 100%, 78% and 73% Bayesian, likelihood and parsimony support, respectively; the clade of strains PD709 and PD711 and the clade of strains PD710 and PD743 with respectively 84 and 98% of the Bayesian posterior probabilities.

With the goal of improving the phylogenetic resolution, we combined the *ACT*, *EF*, *GPD* and *TS* datasets into a single alignment for combined analysis. We did not include the ITS dataset since for *V. longisporum*, the ITS phylogeny does not retrace the evolution of that species [27]. The resulting combined four-locus alignment comprised 77 taxa and 2658 characters, and was submitted to TreeBase (http://purl.org/phylo/treebase/phylows/study/TB2:S11756). There were a total of 32 unique multilocus haplotypes (Table 1). The Bayesian consensus tree is illustrated in Figure 1, it was congruent with the most likely tree (−ln likelihood = 12816.48) and with the 48 most parsimonious trees (Table 1) that differed at poorly supported branches within *V. dahliae* and the outgroup *Gibellulopsis nigrescens* (data not shown, but see support values in Figure 1).

We analyzed the four single-locus datasets jointly despite topological conflicts between them (Figures S2, S3, S4, S5). To evaluate whether single-locus datasets should be concatenated for combined analyses, a conditional combinability approach is often used which states that datasets should not be combined if there are significant differences between them [28], [29]. There is no agreement how much the single-locus datasets are allowed to differ, but topological differences supported by 70–90% of the bootstrap replicates have been used as cutoffs [28], [30]. In our case, there were topological differences supported by up to 100% of the parsimony bootstrap replicates between the single-locus datasets, involving the positions of *V. nubilum*, Species A1 and *V. zaregamsianum*. However, we found that the four-locus phylogeny comprised 35 groups with >70% support, higher than any of the single-locus trees (Table 1). Also, for *V. nubilum*, Species A1 and *V. zaregamsianum*, the combined analyses resulted for each species in the topology that had strongest overall support from the single-locus phylogenies. But the phylogenetic affinities of *V. nubilum* and Species A1 remain uncertain (Figure 1), and more data is needed to conclusively determine the closest relatives of these two species in *Verticillium*. In the combined analyses and in all single-locus datasets but the *EF* dataset, *V. zaregamsianum* formed a well-supported clade with *V. isaacii*, *V. klebahnii* and *V. tricorpus* (Figure 1). One possibility that could explain this divergence is origin by horizontal transfer of the *EF* gene in *V. zaregamsianum*. In conclusion, combined analysis of the single-locus datasets generated a phylogeny with higher overall support than any of the single-locus phylogenies, but did not conclusively settle the phylogenetic positions of *V. nubilum* and Species A1.

### Phylogenetic groups obtained

We were able to infer a robust phylogenetic tree of *Verticillium*. The majority of the branches received maximal support, species were distinct and well defined, and the relationships between species were generally well resolved. As expected, branches with lower support were mainly present within species [31].

We recognized ten different species based on a phylogenetic species concept [31]. Except for the diploid hybrid *V. longisporum* [27], species were defined as terminal or subterminal clades receiving maximal support in the phylogenetic analyses based on the combined four-locus dataset (Figure 1). Each of the nine species level clades contained a single ex-type strain representing herbarium material, and thus linking each clade to one of the following nine species names (Figure 1). *Verticillium albo-atrum*, *V. alfalfae*, *V. dahliae*, *V. isaacii*, *V. klebahnii*, *V. nonalflalfae*, *V. nubilum*, *V. tricorpus* and *V. zaregamsianum*. Alleles of the diploid hybrid *V. longisporum* were present in the three different clades Species A1, Species D1 and *V. dahliae* (Figure 1), reflecting the hybrid origin of *V. longisporum* [27]. *Verticillium longisporum* evolved at least three different times from four different parental lineages in Species A1, Species D1 and *V. dahliae* [27]. Species A1 and Species D1 were not linked to any type material and could not be officially described, since Species A1 and Species D1 have never been found [27].

The evolutionary relationships among the *Verticillium* species was overall well resolved, the species fell into two major clades reflecting morphological similarity. The major clades were Clade Flavexudans containing species producing yellow-pigmented hyphae including *V. albo-atrum*, *V. isaacii*, *V. klebahnii*, *V. tricorpus* and *V. zaregamsianum*, and Clade Flavnonexudans with species devoid of yellow-pigmented hyphae, including *V. alfalfae*, *V. dahliae*, *V. nonalfalfae*, and *V. longisporum* (Figure 1). The exception was *V. nubilum* whose placement in Clade Flavnonexudans agreed with morphological data, but was only supported in the parsimony analyses (Figure 1). The other exception was the position of the *V. longisporum* ancestor Species A1 whose placement in the Bayesian consensus tree (Figure 1) contradicted phylogenetic analyses by Inderbitzin et al. [27] who used a different dataset.

### Mating type distribution in *V. alfalfae* and *V. nonalfalfae*

**Figure 2:**
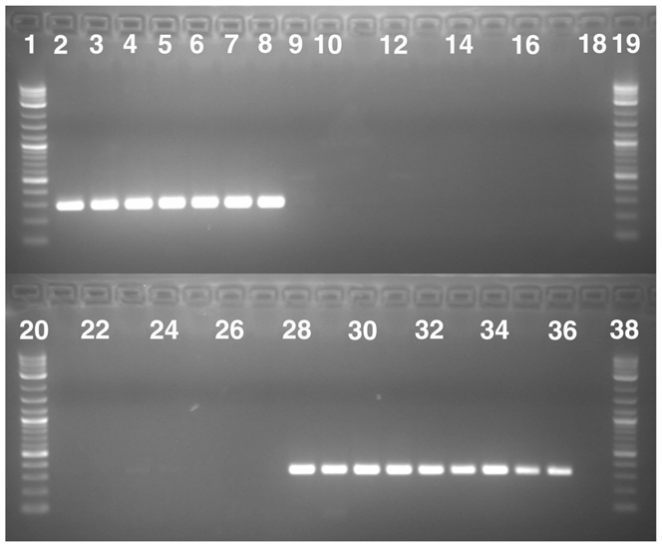
PCR gels documenting the results of the mating type PCR screens of *Verticillium alfalfae* and *V. nonalfalfae* using *MAT1-1* and *MAT1-2* specific primers. Lanes 1–19: *MAT1-1* specific PCR assay using primer set Alf/MAT11r. Lanes 1, 19: 2-log DNA ladders. Lane 18: Negative control. Lanes 2–17: *V. alfalfae* isolates followed by *V. nonalfalfae* isolates, in the same order as listed in Table S1. Lanes 20–38: *MAT1-2* specific PCR assay using primer set HMG21f/MAT21r. Lanes 20, 38: 2-log DNA ladders. Lane 37: Negative control. Lanes 21–36: *V. alfalfae* isolates followed by *V. nonalfalfae* isolates, in the same order as listed in Table S1.

All seven *V. alfalfae* and nine *V. nonalfalfae* isolates were screened for presence of *MAT1-1* and *MAT1-2* idiomorphs which are the two mating compatibility alleles in ascomycetes [32]. All *V. alfalfae* isolates showed the *MAT1-1* specific PCR band whereas all *V. nonalfalfae* isolates lacked that band. All *V. alfalfae* isolates lacked the *MAT1-2* specific band, whereas the *MAT1-2* specific band was present in all *V. nonalfalfae* isolates (Figure 2). Thus, all *V. alfalfae* isolates likely have *MAT1-1* idiomorphs whereas *V. nonalfalfae* isolates have *MAT1-2* idiomorphs.

### Taxonomy

**Figure 3:**
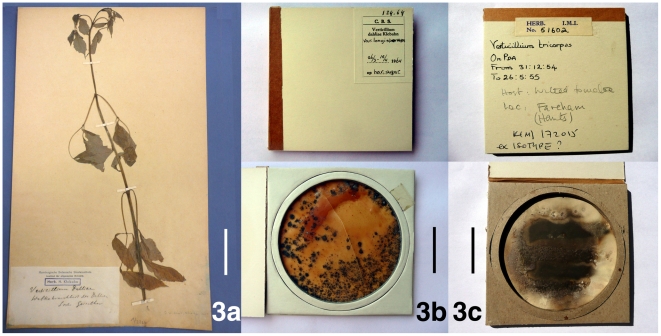
Type material examined in this study. 3a. Holotype specimen of *Verticillium dahliae* from HBG comprising a stem of *Dahlia* sp. cv. Geiselher infected with *V. dahliae*. 3b. Holotype specimen of *V. longisporum* (CBS H-19247) consisting of a dried agar culture (bottom) mounted in a cardboard sleeve (top). The number ‘19247’ was written on an envelope the specimen was placed in (not shown). 3c. Lectotype specimen of *V. tricorpus* (K(M) 172015, IMI 51602) comprising a dried agar culture (bottom) mounted in a cardboard sleeve (top). Scale bar: 3a = 8 cm; 3b, 3c = 3 cm.

The genus *Verticillium* sensu stricto corresponds to a monophyletic group of taxa comprising *V. dahliae* that has been conserved as the type of *Verticillium* [15], [16]. We recognize ten species in *Verticillium* sensu stricto that are listed below in alphabetic order. The information provided for each species was obtained from morphological examination of cultures and herbarium specimens (Figure 3), literature surveys and phylogenetic analyses (Figure 1).

**Figure 4:**
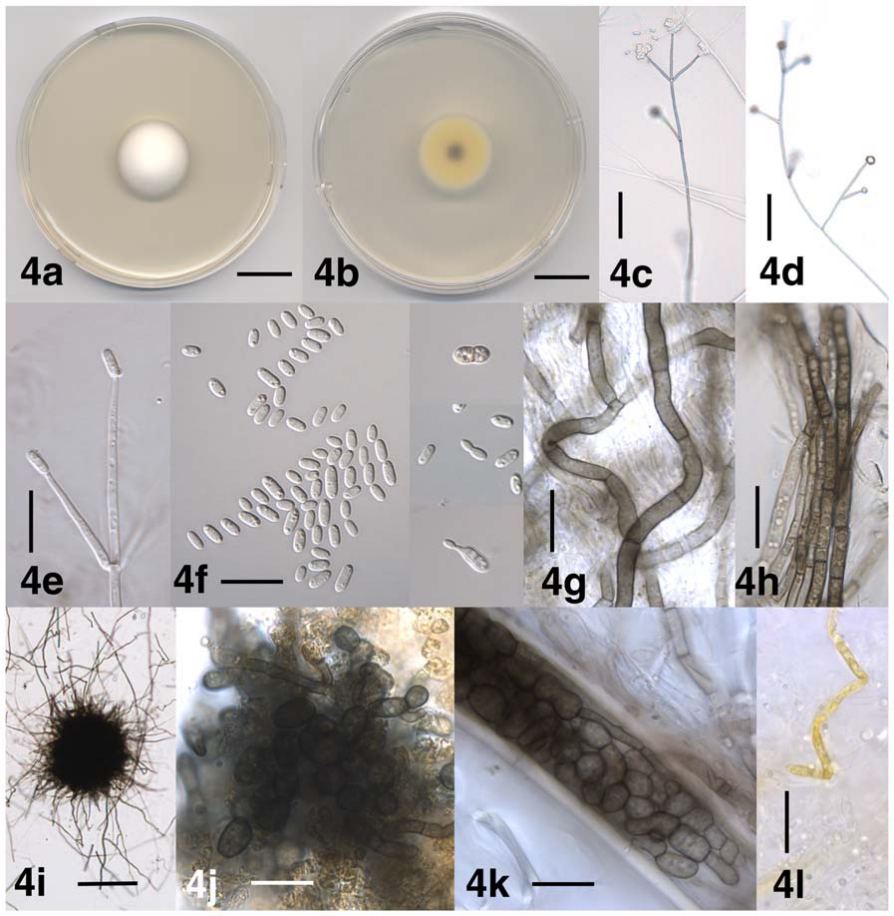
Morphological features of *Verticillium albo-atrum*. 4a. Colony of strain PD747 after 10 days on PDA, frontal view. 4b. Colony of strain PD747 after 10 days on PDA, reverse view. 4c. Conidiophore of strain PD748 after 29 days on WA-p. 4d. Branched conidiophore of strain PD670 after 29 days on WA-p. 4e. Phialide of strain PD670 after 29 days on PDA. 4f. Conidia of strain PD670 after 29 days on PDA; Insets: Pigmented, septate and constricted conidium of strain PD670 after 29 days on PDA, budding conidium and conidium germinating by formation of a phialide, both of strain PD748 after 29 days on WA-p. 4g. Resting mycelium of strain PD747 after 33 days on WA-p. 4h. Aggregated hyphae of resting mycelium in strain PD670 after 28 on WA-p. 4i. Microsclerotium of strain PD670 after 47 days on PLYA. 4j. Microsclerotium of strain PD670 after 28 on WA-p. 4k. Microsclerotium of strain PD747 formed in the lumen of a thick-walled plant cell after 32 days on WA-p. 4l. Hypha of strain PD747 containing yellow pigment after 10 days on PDA. Scale bar: 4a, 4b = 2 cm; 4c, 4d = 50 µm; 4e–4h, 4j–4l = 20 µm; 4i = 100 µm. Imaging method: 4a, 4b = DS; 4c, 4d, 4g–4l = BF; 4e, 4f = DIC.

***Verticillium albo-atrum*** **Reinke & Berthold, Untersuchungen aus dem Botanischen Laboratorium der Universität Göttingen 1:75 (1879)** **Figure 4**

MycoBank: MB199278 (as *V. alboatrum*)

#### Description

Colonies on PDA after two weeks 4.5–5.5 cm diam, white at first, later turning yellow to orange due to the formation of yellow-pigmented hyphae, then darkening due to formation of resting mycelium immersed in the agar medium (Figures 4a, 4b). Aerial mycelium generally abundant, floccose to pruinose, hyphae smooth-walled, (1–) 1.5–4 µm wide. Conidiophores erect or slanted, generally determinate (Figure 4c), branched or unbranched (Figure 4d), formed disjointedly throughout the colonies, hyaline, base brown-pigmented at times, 80–480 µm in length, 3–6 µm wide, narrowing towards the apex to 2–2.5 µm, transversely septate, septa spaced more narrowly towards the apex. Conidiogenous cells are phialides, arranged in 1–4 (–6) whorls along conidiophores (Figures 4c, 4d), arising below transverse septum (Figure 4e). Whorls spaced 20–140 µm apart, closer towards the apex, consisting of (1–) 2–4 (–6) phialides (Figures 4c, 4d, 4e). Apical whorls consisting of one apical and one to several lateral phialides (Figures 4c, 4d, 4e). Phialides subulate, tapering from 1.5–3 µm at the base to 1–1.5 µm at the tip, terminal phialides 40–80 µm long, lateral phialides 20–50 µm long (Figure 4e). Conidia hyaline, smooth-walled, cylindrical with rounded apices to oval (Figure 4f), tapering at times, (3.0–) 6.0 µm±1.5 µm (–10.5)×(2.0–) 3.0 µm±0.5 µm (–6.0) (l/w = (1.1–) 2.0±0.4 (–3.0), n = 86), accumulating at the tip of the phialides (Figures 4c, 4d). After 4 wks, a small number of conidia (generally <1%) with central septum, constricted at the septum at times (Figure 4f). Budding conidia and conidia germinating by formation of a phialide observed (Figure 4f). Resting mycelium present, consisting of brown-pigmented hyphae, up to 7 µm wide, thick-walled, straight or curved, solitary or aggregated, up to 25 µm wide (Figures 4g, 4h). Microsclerotia present, composed of tightly interwoven, torulose brown-pigmented hyphae, rounded or variously shaped, up to 230 µm diam and consisting of rounded to elongate cells, up to 10 µm diam (Figures 4i, 4j, 4k). Yellow-pigmented hyphal cells present at times, up to 5 µm wide (Figure 4l).

#### Types

**Figure 5:**
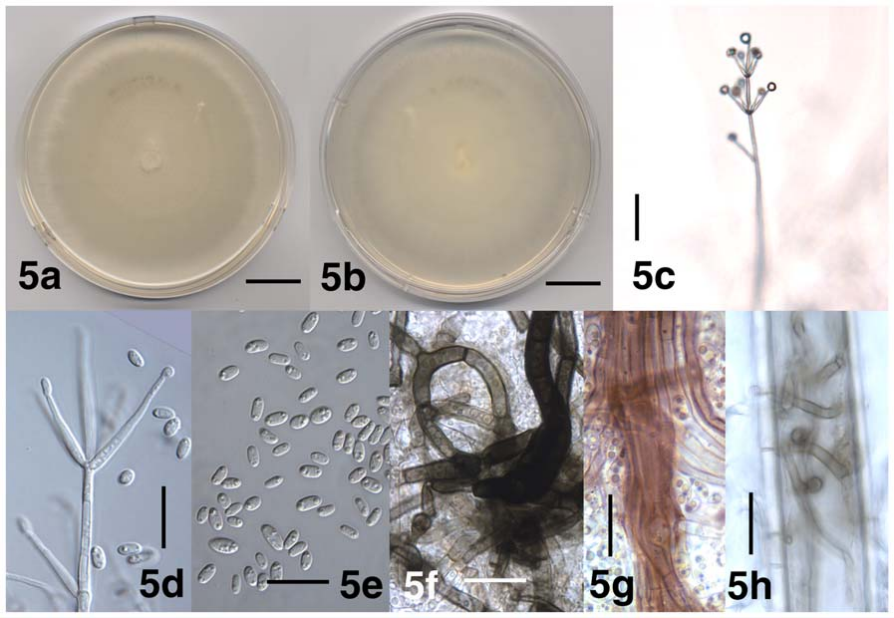
Morphological features of *Verticillium alfalfae*. 5a. Colony of strain PD682 after 24 days on PDA, frontal view. 5b. Colony of strain PD682 after 24 days on PDA, reverse view. 5c. Conidiophore of strain PD682 after 31 days on WA-p. 5d. Phialide of strain PD489 after 30 days on WA-p. 5e. Conidia of strain PD682 after 30 days on WA-p. 5f. Resting mycelium of strain PD489 after 30 days on WA-p. 5g. Aggregated hyphae of resting mycelium in strain PD682 after 73 days on PDA. 5h. Resting mycelium of strain PD683 in the lumen of a thick-walled plant cell after 32 days on WA-p. Scale bar: 5a, 5b = 2 cm; 5c = 50 µm; 5d–5h = 20 µm. Imaging method: 5a, 5b = DS; 5c, 5f–5h = BF; 5d, 5e = DIC.

**Figure 6:**
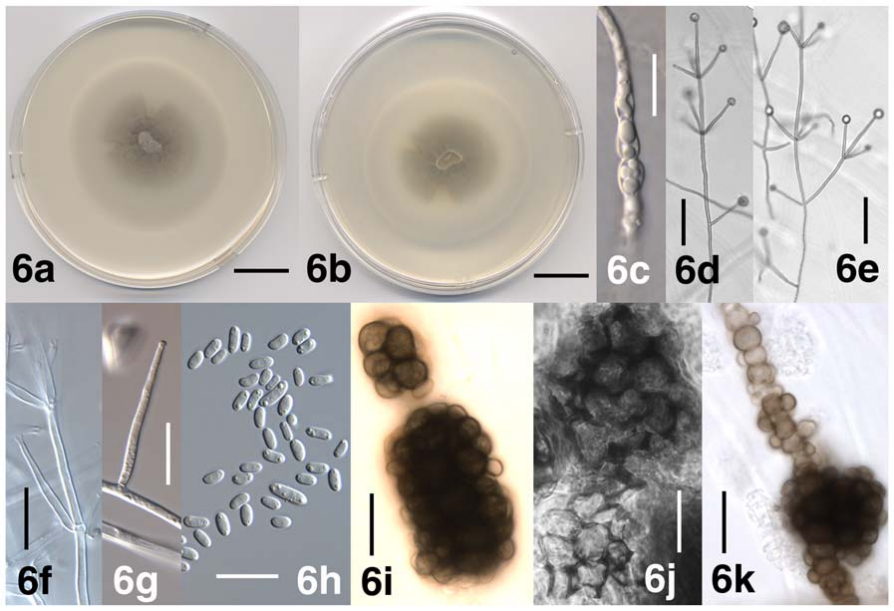
Morphological features of *Verticillium dahliae* strain PD322 (ex-epitype) unless otherwise noted. 6a. Colony after 14 days on PDA, frontal view. 6b. Colony after 14 days on PDA, reverse view. 6c. Inflated cells present in mycelium after 28 days on PDA. 6d. Conidiophore after 15 days on WA-p. 6e. Branched conidiophore after 12 days on WA-p. 6f. Whorl phialide after 25 days on WA-p. 6g. Solitary phialide after 14 days on PDA. 6h. Conidia after 9 days on PDA. 6i. Microsclerotia after 12 days on WA-p. 6j. Microsclerotia of the *V. dahliae* holotype material from stem of *Dahlia* sp. 6k. Short brown-pigmented hypha composed of torulose cells attached to microsclerotium after 49 days on PDA. Scale bar: 6a, 6b = 2 cm; 6c, 6f–6k = 20 µm; 6d, 6e = 50 µm. Imaging method: 6a, 6b = DS; 6c, 6f–6h = DIC; 6d, 6e, 6i, 6k = BF; 6j = PC.

**Figure 7:**
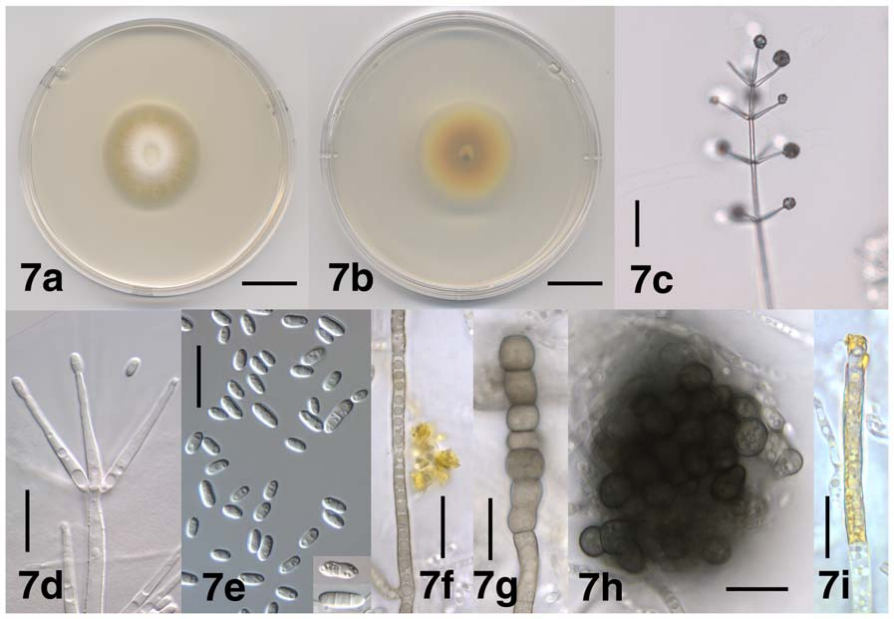
Morphological features of *Verticillium isaacii*. 7a. Colony of strain PD619 after 10 days on PDA, frontal view. 7b. Colony of strain PD619 after 10 days on PDA, reverse view. 7c. Conidiophore of strain PD618 after 21 days on WA-p. 7d. Phialides of strain PD660 as part of an apical whorl after 21 days on WA-p. 7e. Conidia of strain PD611 after 22 days on WA-p; Insets: One-septate, constricted conidium and two septate conidium of strain PD660 after 21 days on WA-p. 7f. Hypha of resting mycelium and yellow crystal of strain PD752 after 35 days on PDA. 7g. Chlamydospores of strain PD752 after 35 days on PDA. 7h. Microsclerotium of strain PD752 after 35 days on PDA. 7i. Hyphal cell of strain PD660 encrusted with yellow pigment after 20 days on PDA. Scale bar: 7a, 7b = 2 cm; 37c = 50 µm; 7d–7i = 20 µm. Imaging method: 7a, 7b = DS; 7c, 7f–7i = BF; 7d, 7e = DIC.

**Figure 8:**
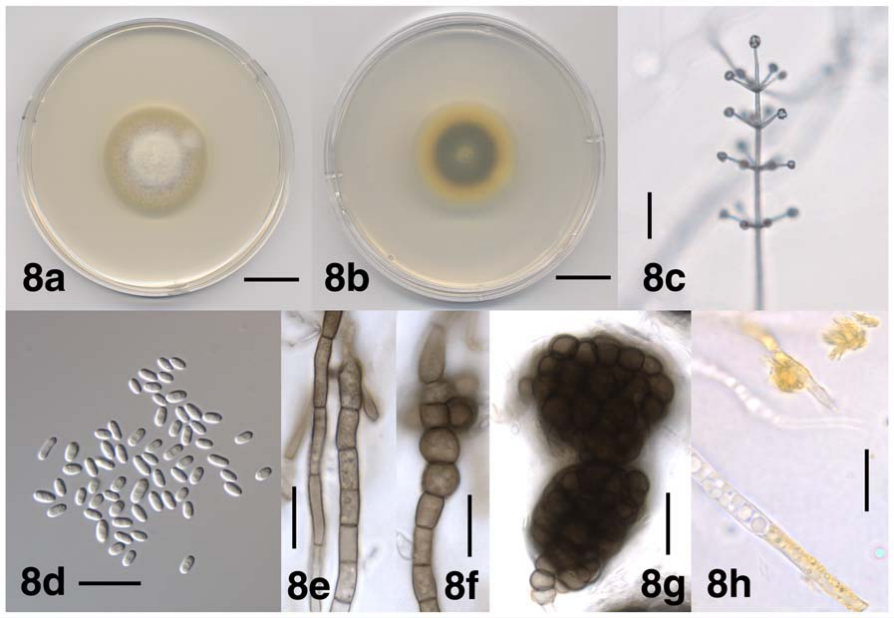
Morphological features of *Verticillium klebahnii*. 8a. Colony of strain PD659 after 10 days on PDA, frontal view. 8b. Colony of strain PD659 after 10 days on PDA, reverse view. 8c. Conidiophore of strain PD659 after 24 days on WA-p. 8d. Conidia of strain PD401 after 35 days on WA-p. 8e. Resting mycelium of strain PD657 after 32 days on WA-p. 8f. Chlamydospores of strain PD657 after 32 days on WA-p. 8g. Microsclerotia of strain PD401 after 32 days on WA-p. 8h. Hyphal cells of strain PD401 with yellow pigment and yellow crystals after 20 days on PDA. Scale bar: 8a, 8b = 2 cm; 8c = 50 µm; 8d–8h = 20 µm. Imaging method: 8a, 8b = DS; 8c, 8e–8h = BF; 8d = DIC.

**Figure 9:**
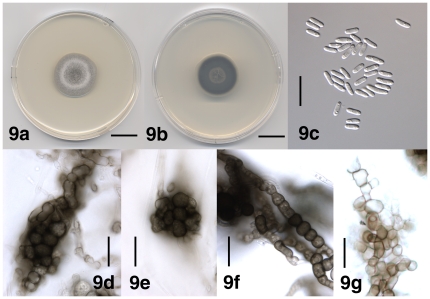
Select morphological features of *Verticillium longisporum*. 9a. Colony of strain PD356 after 10 days on PDA, frontal view. 9b. Colony of strain PD356 after 10 days on PDA, frontal view. 9c. Conidia of strain PD348 after 35 days on PDA. 9d. Elongate microsclerotium of strain PD356 after 35 days on PDA. 9e. Rounded microsclerotium of strain PD356 after 35 days on PDA. 9f. Short brown-pigmented hyphae attached to microsclerotium in strain PD348 after 35 days on PDA 9g. Elongate microsclerotium from *V. longisporum* holotype specimen CBS H-19247. Scale bar: 9a, 9b = 1 cm; 9c–9g = 20 µm; Imaging method: 9a, 9b, = DS; 9c = DIC; 9d–9g = BF.

**Figure 10:**
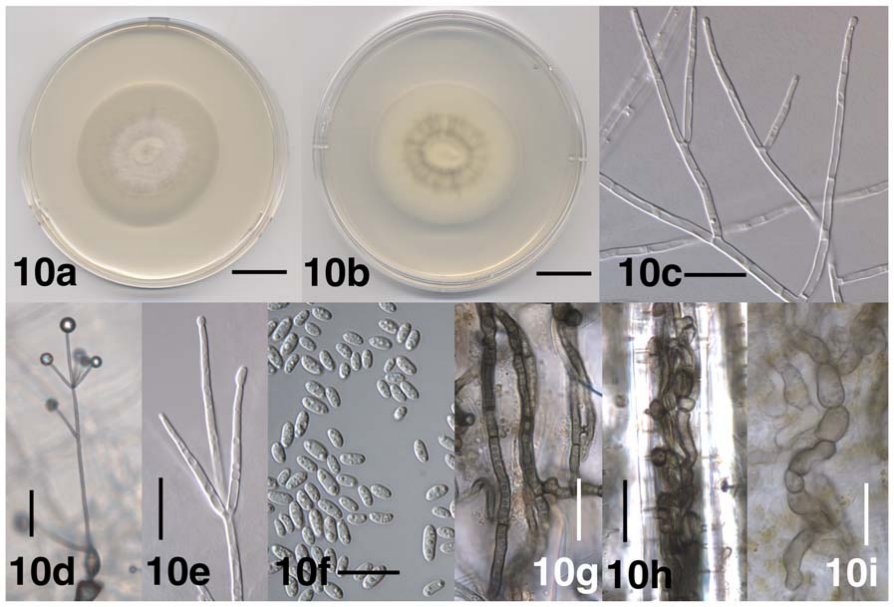
Morphological features of *Verticillium nonalfalfae*. 10a. Colony of strain PD592 after 14 days on PDA, frontal view. 10b. Colony of strain PD592 after 14 days on PDA, reverse view. 10c. Branched conidiophore of strain PD616 after 13 days on WA-p. 10d. Conidiophore of strain PD616 after 13 days on WA-p. 10e. Phialide of apical whorl of strain PD616 after 13 days on WA-p. 10f. Conidia of strain PD808 after 31 days on WA-p. 10g. Resting mycelium of strain PD811 after 18 days on WA-p. 10h. Intertwined hyphae of resting mycelium in strain PD810 in the lumen of a thick-walled plant cell after 18 days on WA-p. 10i. Torulose hyphal cells of resting mycelium in strain PD592 after 18 days on WA-p. Scale bar: 10a, 10b = 2 cm; 10c, 10e–10i = 20 µm; 10d = 50 µm. Imaging method: 10a, 10b = DS; 10c, 10e, 10f = DIC; 10d, 10g–10i = BF.

**Figure 11:**
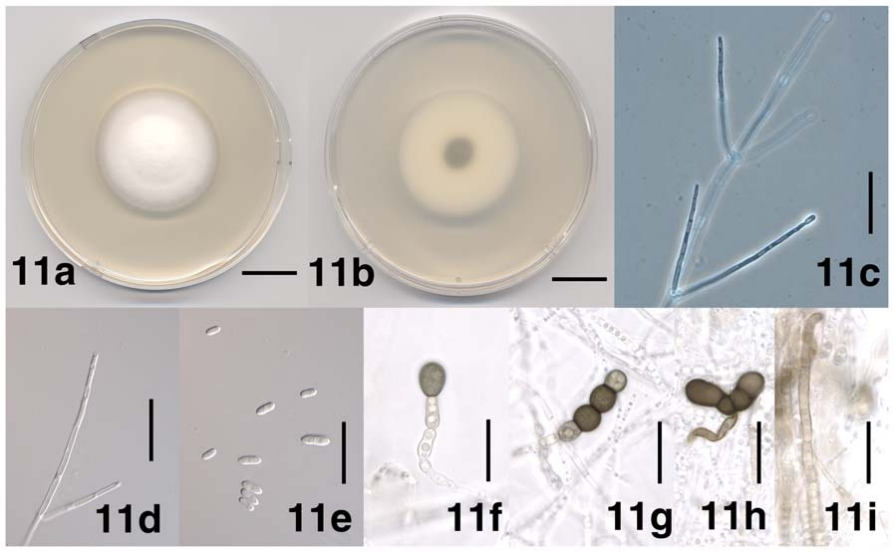
Morphological features of *Verticillium nubilum*. **11.** Colony of strain P742 after 13 days on PDA, frontal view. 1b. Colony of strain PD742 after 13 days on PDA, reverse view. 11c. Conidiophore of strain PD621 after 17 days on WA-p. 11d. Apical phialide of strain PD621 after 17 days on WA-p. 11e. Conidia of strain PD621 after 17 days on WA-p. 11f. Solitary chlamydospore of strain PD742 after 17 days on WA-p. 11g. Linear chain of chlamydospores of strain PD742 after 17 days on WA-p. 11h. Angular chain of chlamydospores of strain PD621 after 25 days on PDA. 11i. Brown-pigmented hyphae of strain PD621 after 25 days on PDA. Scale bar: 11a, 11b = 1 cm; 11c–11i = 20 µm; Imaging method: 11a, 11b, = DS; 11c = PC; 11d, 11e = DIC; 11f–11i = BF.

Holotype: Missing, not found at GOET, B, M; Lectotype (designated herein): Illustrations from protolog: Figures of Plate (‘Tafel’) 8 and Figures 1, 2, 3, 4, 5, 6, 7, 8, 9, 10, 11 of Plate 9 in Reinke and Berthold [21], available online at http://books.google.com/books?id=iWgVAAAAYAAJ&dq=Die%20Kr%C3%A4uselkrankheit%20der%20Kartoffel&pg=PA107#v=onepage&q=Die%20Kr%C3%A4uselkrankheit%20der%20Kartoffel&f=false (accessed on October 5, 2011); Epitype (designated herein): Dried culture of *Verticillium albo-atrum* strain PD747 (Canada: Prince Edward Island; potato field soil) deposited at UC (UC 1953892), an ex-epitype culture at CBS (CBS 130340) and NRRL (NRRL 54797).

#### Specimens examined

The description was based on *Verticillium albo-atrum* strains PD670 (USA: WI; Irish potato), PD693 (UK; Irish potato), PD746 (Canada: New Brunswick; potato field soil), PD747 (Canada: Prince Edward Island; potato field soil) and PD748 (Canada: Prince Edward Island; potato field soil)(Table S1).

#### Distribution and host range

Currently known from Canada, Germany, UK and USA (WI). Substrates include Irish potato and soil collected from Irish potato fields.

#### Commentary

*Verticillium albo-atrum* was described by Reinke and Berthold in 1879 from diseased potato plants collected near Göttingen, Germany [21]. The protolog of *V. albo-atrum* contains detailed descriptions and drawings, but no reference is made to type material. We inquired at the herbaria of Göttingen (GOET), Berlin (B) and München (M), none of which has any *V. albo-atrum* type material or any other *V. albo-atrum* material deposited by Reinke and Berthold. According to Klebahn [33], original cultures of *V. albo-atrum* are no longer available. We did not find any *V. albo-atrum* cultures by Reinke and Berthold in any of the major culture collections (CBS, IMI, DSMZ, ATCC). Thus, in absence of any original fungal material, we designated the illustrations from the *V. albo-atrum* protolog in Plate 8 (Figures 1, 2, 3, 4, 5, 6, 7, 8) and Plate 9 (Figures 1, 2, 3, 4, 5, 6, 7, 8, 9, 10, 11) in Reinke and Berthold [21] as the lectotype for *V. albo-atrum*. According to The International Code of Botanical Nomenclature (ICBN) [34] this is permissible when the holotype, all cited or uncited original specimens and all original cultures are missing (Art. 8.4, Art. 9.2, Art. 9.10, Art. 37.4). To serve as an interpretive type, we designated a *V. albo-atrum* epitype with an ex-epitype culture for molecular studies. Designation of an epitype is permissible according to ICBN when serving the precise application of a name (Article 9.7).

The original description of *V. albo-atrum* by Reinke and Berthold [21] was based on observations from decaying potato stems, and is congruent with our observations from the *V. albo-atrum* isolates examined in this study. The exception was the presence of yellow pigment associated with hyphal cells (Figure 4l) not seen by Reinke and Berthold [21]. However, Klebahn [35] reported that *V. albo-atrum* mycelium on Salep Agar medium was white with a yellow tinge (p. 64), whereas *V. dahliae* mycelium was described as white (p. 65).

In addition to resting mycelium, *Verticillium albo-atrum* also forms microsclerotia (Figures 4i, 4j, 4k). Microsclerotia are very ‘small, firm, frequently rounded masses of hyphae with or without the addition of host tissue or soil.’ [36]. The *V. albo-atrum* microsclerotia were described and illustrated in the protolog on pages 74 and 75, and in Figures 1 and 2 of Plate 9 [21], a translation from the German original is provided by Isaac [22]. The microsclerotia consist of aggregations of brown-pigmented, thick-walled hyphae, no lateral cell divisions are involved in their formation [21]. This is opposed to the microsclerotia of *V. dahliae* where an increase in width is achieved by the lateral divisions of hyphal cells as described by Klebahn [35] on pages 56 and 57, and illustrated in Klebahn's [35] Figure 8. Microsclerotia were only observed on WA-p and PLYA media, they were absent from strains cultured on PDA. *Verticillium albo-atrum* has frequently been confused with *V. alfalfae* and *V. nonalfalfae* that form resting mycelium but no microsclerotia.

The name ‘*V. albo-atrum*’ is correct with or without hyphen (Art. 23.1), the hyphenated form is more commonly encountered in the literature.

***Verticillium alfalfae*** **Inderb., H. W. Platt, R. M. Bostock, R. M. Davis & K. V. Subbarao,** ***sp. nov.*** **Figure 5**

MycoBank: MB563552

Etymology: *Medicago sativa* (‘alfalfa’), the only currently known host of this species.

#### Latin diagnosis

Verticillio nonalfalfae morphologia simile, sed characteribus sequentiarum nucleidearum distinguendum. Actin positione 21 (T), 72 (G), 78 (T), 459 (A), 462 (A); Elongation factor 1-alpha positione 149 (G), 157 (G), 175 (G), 225 (A), 265 (T), 266 (A), 271 (A), 280 (C), 304 (T), 346 (C), 428 (T), 429 (T), 441 (G), 465 (T), 469 (C), 474 (T), 591 (C), 596 (T), 600 (C), 624 (T); Glyceraldehyde-3-phosphate dehydrogenase positione 173 (A), 324 (C); Tryptophan synthase positione 87 (A), 161 (T), 169 (C), 246 (T), 273 (T), 315 (T), 583 (T), 601 (C).

#### Description

Colonies on PDA after two weeks 3.5–4.5 cm diam, white at first (Figures 5a, 5b), later darkening due to the formation of resting mycelium immersed in the agar. Aerial mycelium generally abundant, floccose to pruinose, hyphae smooth-walled, 2–3 µm wide. Conidiophores erect or slanted, generally determinate (Figure 5c), branched or unbranched, formed disjointedly throughout the colonies, hyaline, base brown-pigmented at times, enlarged to up to 11 µm at times, 70–570 µm in length, 4.5–6.5 µm wide, narrowing towards the apex to 2–2.5 µm, transversely septate, septa spaced more narrowly towards the apex (Figure 5c). Conidiogenous cells are phialides (Figure 5d), arranged in 1–4 (–6) whorls along conidiophores (Figure 5c). Whorls spaced 30–130 µm apart, closer towards the apex, consisting of (1–) 2–5 (–6) phialides, arising below transverse septum (Figures 5c, 5d). Apical whorls consisting of one apical and one to several lateral phialides (Figures 5c, 5d). Phialides subulate, tapering from 2–3 µm at the base to 1–2.5 µm at the tip, terminal phialides 40–60 µm long, lateral phialides 20–40 µm long (Figure 5d). Conidia hyaline, smooth-walled, cylindrical with rounded apices to oval (Figure 5e), allantoid at times, (4.5–) 6.0 µm±1.0 µm (–11.0)×(2.5–) 3.0 µm±0.5 µm (–4.0) (l/w = (1.4–) 1.9±0.3 (–2.9), n = 68), accumulating at the tip of the phialides (Figure 5c). Resting mycelium present (Figures 5f, 5g, 5h), consisting of brown-pigmented hyphae, up to 9 µm wide, thick-walled (Figures 5f, 5g), straight or curved, solitary or aggregated (Figure 5g), torulose at times.

#### Types

Holotype: Dried culture of *V. alfalfae* strain PD489 (USA; alfalfa) deposited at UC (UC 1953895), an ex-holotype culture at CBS (CBS 130603) and NRRL (NRRL 54790).

#### Specimens examined

The description was based on *V. alfalfae* strains PD338 (USA: PA; alfalfa), PD353 (USA: PA; alfalfa), PD489 (USA; alfalfa), PD620 (Canada; alfalfa), PD681 (USA; alfalfa), PD682 and PD683 (Japan: Hokkaidou; alfalfa) (Table S1).

#### Distribution and host range

Currently known from Canada, Japan and the USA (PA), only from alfalfa.

#### Commentary

*Verticillium alfalfae* is morphologically indistinguishable from *V. nonalfalfae*.

***Verticillium dahliae*** **Kleb., Mycologisches Centralblatt 3: 66 (1913)** **Figures 3a** **,** **6**

MycoBank: MB196942

#### Description

Colonies on PDA after two weeks 4–6 cm diam, white at first, later darkening due to the formation of microsclerotia (Figures 6a, 6b). Aerial mycelium generally abundant, floccose, at times sparse and pruinose, or appressed to the agar and appearing water-soaked. Aerial hyphae smooth-walled, (1.5–) 2–4 µm wide, at times containing inflated cells up to 9 µm wide (Figure 6c). Conidiophores erect or slanted, generally determinate (Figure 6d), branched or unbranched (Figure 6e), formed disjointedly throughout the colonies, hyaline, 80–800 µm in length, 3–4 µm wide, narrowing towards the apex, transversely septate, septa spaced more narrowly towards the apex. Conidiogenous cells are phialides (Figure 6f), arranged in (1–) 2–3 (–10) whorls along conidiophores (Figures 6d, 6e), arising below transverse septum (Figure 6f). Whorls spaced 50–100 µm apart, closer towards the apex, consisting of (1–) 2–4 (–6) phialides (Figures 6d, 6e). Apical whorls consisting of one apical and one to several lateral phialides (Figure 6d). At times solitary phialides are formed laterally from vegetative hyphae (Figure 6g). Phialides subulate, tapering from 2–3 µm at the base to 1–2 µm at the tip, terminal phialides 40–60 µm long, lateral phialides 25–50 µm long (Figures 6d, 6e, 6f, 6g). Conidia hyaline, smooth-walled, non-septate, cylindrical with rounded apices to oval (Figure 6h), allantoid or tapering at times, (3.5–) 6.5 µm±1.5 µm (–13.5)×(2.0–) 3.0 µm±0.5 µm (–4.5) (l/w = (1.4–) 2.2±0.3 (–3.4), n = 80), accumulating at the tip of the phialides (Figures 6d, 6e). Microsclerotia immersed in agar, regularly or irregularly distributed throughout the colonies, composed of rounded, brown-pigmented cells up to 13 µm diam, solitary microsclerotia rounded to elongate or irregular in shape, 25–100 µm diam, aggregates of microsclerotia up to 200 µm diam (Figures 6i, 6j, 6k). At times short, brown-pigmented hyphae attached to microsclerotia present (Figure 6k).

#### Types

Holotype: Specimen *V. dahliae* (Germany; *Dahlia* sp.) at HBG (Figures 3a, 6j) [16]; Epitype (designated herein): Dried culture of *Verticillium dahliae* strain PD322 (USA: CA, lettuce) deposited at UC (UC 1953893), an ex-epitype culture at CBS (CBS 130341) and NRRL (NRRL 54785).

#### Specimens examined

The description was based on *Verticillium dahliae* strains PD322 (USA: CA; lettuce), PD327 (USA: CA; bell pepper) and PD502 (USA: WI; maple) (Table S1). The *V. dahliae* holotype specimen was also examined (Figures 3a, 6j).

#### Distribution and host range

Currently known from Brazil, Canada, Denmark, France, Germany, Iran, Israel, Italy, Japan, Netherlands, Russia, Spain, Sweden, UK, Ukraine, and USA (CA, ID, IL, OR, TX, WA, WI) [27]. Substrates include Anaheim pepper, annual sunflower, apricot, ash, bell pepper, cabbage, celandine, chili pepper, common flax, eggplant, European smoketree, garden tomato, globe artichoke, horseradish, hybrid strawberry, Icelandic poppy, Irish potato, jalapeno, Japanese maple, lettuce, maple, olive, opium poppy, paprika, pepper, peppermint, pistachio nut, purple coneflower, rape, scentless false mayweed, spinach, stock, sweet almond, udo, upland cotton, and watermelon [27] that represent fourteen different plant families (Aceraceae, Amaranthaceae, Anacardiaceae, Araliaceae, Asteraceae, Brassicaceae, Cucurbitaceae, Fabaceae, Linaceae, Malvaceae, Oleaceae, Papaveraceae, Rosaceae, Solanaceae).

#### Commentary

*Verticillium dahliae* is the type of *Verticillium* and was described by Klebahn [35] from *Dahlia* sp. cv. Geiselher in Germany (Figure 3a). *Verticillium dahliae* is not the oldest species of the genus, but it has the largest impact as a pathogen, is common and genetically relatively homogenous, and has thus been conserved as the type of the genus [16], [34]. Since a viable ex-holotype culture is no longer available [33], and DNA extraction attempts from the holotype specimen failed, we designated a *V. dahliae* epitype with an ex-epitype culture that serves as an interpretive type for molecular studies.

The original description of *V. dahliae* by Klebahn [35] was based on material from *Dahlia* sp., and from cultures on Salep Agar medium which is a mixture of polysaccharides contained in orchid tubers [37]. The composition of Klebahn's medium is unknown, but as a reference, Noël [38] isolated fungal symbionts of orchids using a clear, weak decoction of salep containing 2% agar. We examined the *V. dahliae* holotype material which contains an approximately 50 cm long stalk of *Dahlia* sp. ‘Sorte Geiselher’ and several leaves (Figure 3a). The microsclerotia present on the stem (Figure 6j) were similar to the microsclerotia formed in culture (Figure 6i). No conidiophores were observed, these are difficult to detect on *Dahlia* sp. [35], but are illustrated as part of the protolog [35].

The description of *V. dahliae* based on *V. dahliae* strains PD322, PD327 and PD502 agreed with the original description by Klebahn [35] except that we failed to detect strands of erect, hyphal aggregates containing conidia and microsclerotia. Klebahn [35] reported the presence of a slightly wider cell (foot cell) at the base of conidiophores. Since foot cells were absent in culture and we did not inoculate live plants, we were unable to confirm the presence of foot cells in *V. dahliae*. The dimensions provided by Klebahn [35] for microsclerotia, conidiophores, conidiogenous cells and conidia were at the lower end of the range of dimensions that we observed. Our dimensions were similar to reports in the literature for conidia [22] and microsclerotia [22], [39], [40], [41]. Short brown-pigmented hyphae attached to microsclerotia were illustrated by Klebahn [35], the ones that we observed resemble immature microsclerotia as illustrated by Klebahn [35] and Isaac [22]. *Verticillium dahliae* resembles *V. longisporum* but has smaller conidia.

***Verticillium isaacii*** **Inderb., R. M. Bostock, R. M. Davis & K. V. Subbarao,** ***sp. nov.*** **Figure 7**

MycoBank: MB563553

Etymology: Named after Ivor Isaac (1914–1978), in recognition of significant contributions to *Verticillium* taxonomy.

#### Latin diagnosis

Verticillio tricorpus morphologia simile, sed characteribus sequentiarum nucleidearum distinguendum. Actin positione 79 (T), 115 (T), 292 (T), 380 (T), 410 (T), 432 (A); Elongation factor 1-alpha positione 142 (C), 162 (A), 166 (T), 185 (A), 190 (T), 230 (A), 235 (G), 248 (A), 260 (A), 331 (A), 363 (T), 366 (G); Glyceraldehyde-3-phosphate dehydrogenase positione 153 (C), 278 (C); Tryptophan synthase positione 133 (A), 143 (A), 383 (G).

#### Description

Colonies on PDA after two weeks 2.5–6 cm diam, white at first, later yellow, reverse orange to yellow, then darkening due to the formation of resting mycelium, chlamydospores and microsclerotia (Figures 7a, 7b). Aerial mycelium generally abundant, floccose, hyphae smooth-walled, 1–3.5 µm wide. Conidiophores erect or slanted (Figure 7c), generally determinate, branched or unbranched, formed disjointedly throughout the colonies, hyaline, verruculose surface ornamentation present at times, 105–690 µm in length, 3–6 µm wide, narrowing towards the apex to 2–2.5 µm, transversely septate, septa spaced more narrowly towards the apex. Conidiogenous cells are phialides (Figure 7d), arranged in (1–) 2–4 (–6) whorls along conidiophores (Figure 7c), arising below transverse septum. Whorls spaced 25–60 µm apart, closer towards the apex, consisting of (1–) 3–5 (–6) phialides (Figure 7c). Apical whorls consisting of one apical and one to several lateral phialides (Figures 7c, 7d). Phialides subulate, tapering from 2–3.5 µm at the base to 1–1.5 µm at the tip, terminal phialides 30–65 µm long, lateral phialides 20–40 µm long (Figure 7d). Conidia hyaline, smooth-walled, cylindrical with rounded apices to oval (Figure 7e), tapering at times, (3.5–) 6.0 µm±1.5 µm (–14.5)×(1.5–) 3.0 µm±0.5 µm (–5.0) (l/w = (1.4–) 1.9±0.3 (–3.5), n = 73), accumulating at the tip of the phialides (Figure 7c). Conidia rarely one- or two-septate, constricted at the septum at times (Figure 7e). Resting mycelium, chlamydospores and microsclerotia present. Resting mycelium consisting of brown-pigmented hyphae, up to 5 µm wide (Figure 7f), chlamydospores solitary or in chains, up to 12 µm wide (Figure 7g), microsclerotia rounded or variously shaped, up to 70 µm diam and consisting of rounded to elongate cells, up to 10 µm wide (Figure 7h). Yellow-pigmented hyphal cells present, up to 5.5 µm wide, containing globules of yellow pigment, at times pigment accumulating as crystals outside the cells, up to 21 µm diam (Figures 7f, 7i).

#### Types

Holotype: Dried culture of *V. isaacii* strain PD660 (USA: CA; lettuce) deposited at UC (UC 1953896), an ex-holotype culture at CBS (CBS 130343) and NRRL (NRRL 54792).

#### Specimens examined

The description was based on *V. isaacii* strains PD341, PD343, PD367, PD437, PD610, PD611, PD612, PD613, and PD660 (USA: CA; lettuce), PD618 (UK; garden tomato), PD619 (Canada; soil), PD661 (USA: WA; lettuce), PD752 and PD753 (USA, WA; spinach) (Table S1).

#### Distribution and host range

Currently known from Canada, UK and USA (CA, WA). Substrates include garden tomato, globe artichoke, hairy nightshade, lettuce, spinach and soil.

#### Commentary

*Verticillium isaacii* is morphologically indistinguishable from *V. klebahnii* and *V. tricorpus*.

***Verticillium klebahnii*** **Inderb., R. M. Bostock, R. M. Davis & K. V. Subbarao,** ***sp. nov.*** **Figure 8**

MycoBank: MB563554

Etymology: Named after Heinrich Klebahn (1859–1942), in recognition of significant contributions to *Verticillium* taxonomy.

#### Latin diagnosis

Verticillio tricorpus morphologia simile, sed characteribus sequentiarum nucleidearum distinguendum. Actin positione 82 (C), 92 (T), 256 (C); Elongation factor 1-alpha positione 160 (C), 186 (T), 191 (A), 195 (T), 196 (G), 203 (A), 215 (A), 220 (A), 264 (C), 312 (C), 352 (G), 355 (C), 363 (G), 384 (C); Tryptophan synthase positione 167 (T).

#### Description

Colonies on PDA after two weeks 4–6.5 cm diam, white at first, later yellow, reverse orange to yellow, then darkening due to the formation of brown-pigmented hyphae, chlamydospores and microsclerotia (Figures 8a, 8b). Aerial mycelium generally abundant, floccose, hyphae smooth-walled, 1–3.5 µm wide. Conidiophores erect or slanted (Figure 8c), generally determinate, branched or unbranched, formed disjointedly throughout the colonies, hyaline, verruculose surface ornamentation present at times, 130–700 µm in length, 3–5 µm wide, narrowing towards the apex to 2–3 µm, transversely septate, septa spaced more narrowly towards the apex. Conidiogenous cells are phialides, arranged in (1–) 2–7 (–8) whorls along conidiophores (Figure 8c). Whorls spaced 30–65 µm apart, closer towards the apex, consisting of (1–) 2–5 (–7) phialides (Figure 8c), arising below transverse septum. Apical whorls consisting of one apical and one to several lateral phialides (Figure 8c). Phialides subulate, tapering from 1.5–2.5 µm at the base to 1–1.5 µm at the tip, terminal phialides 30–60 µm long, lateral phialides 18–45 µm long (Figure 8c). Conidia hyaline, smooth-walled, cylindrical with rounded apices to oval (Figure 8d), tapering at times, (3.5–) 5.0 µm±0.5 µm (–10.0)×(1.5–) 2.5 µm±0.5 µm (–4.5) (l/w = (1.0–) 1.9±0.2 (–2.4), n = 73), accumulating at the tip of the phialides (Figure 8c). Resting mycelium, chlamydospores and microsclerotia present. Resting mycelium consisting of brown-pigmented hyphae, up to 8 µm wide (Figure 8e), chlamydospores solitary or in chains, up to 13 µm wide (Figure 8f), microsclerotia rounded or variously shaped, up to 80 µm diam and consisting of rounded to elongate cells, up to 9 µm wide (Figure 8g). Yellow-pigmented hyphal cells present, up to 7.5 µm wide, containing globules of yellow pigment, at times pigment accumulating as crystals outside the cell, up to 14 µm diam (Figure 8h).

#### Types

Holotype: Dried culture of *V. klebahnii* strain PD401 (USA: CA; lettuce) deposited at UC (UC 1953897), an ex-holotype culture at CBS (CBS 130344) and NRRL (NRRL 54789).

#### Specimens examined

The description was based on *V. klebahnii* strains PD347, PD401, PD407 and PD458 (USA: CA; lettuce), PD657, PD658 and PD659 (USA: WA; lettuce) (Table S1).

#### Distribution and host range

Currently only known from the USA (CA, WA) from lettuce.

#### Commentary

*Verticillium klebahnii* is morphologically indistinguishable from *V. isaacii* and *V. tricorpus*. *Verticillium isaacii* and *V. klebahnii* were described as new species because no synonyms of the morphologically similar *V. tricorpus* were available (www.indexfungorum.org, accessed on September 30, 2011).

***Verticillium longisporum*** **(C. Stark) Karapapa, Bainbr. & Heale, Mycological Research 101(11): 1293 (1997)** **Figures 3b** **,** **9**

Basionym: *Verticillium dahliae* var. *longisporum* C. Stark, Gartenbauwissenschaft 26(8): 508 (1961)

MycoBank: MB443108

#### Description

*Verticillium longisporum* was described by Stark [42] and in more detail by Karapapa et al. [24]. We documented colony morphology (Figures 9a, 9b), conidia (Figure 9c) and microsclerotia (Figures 9d, 9e, 9f, 9g). We measured microsclerotia and conidia, and assessed the number of phialides per whorl. Microsclerotia were rounded to elongate, 37–240×25–52 µm (Figures 9d, 9e). Conidia were (5.5–) 8.5 µm±2.5 µm (–15.0)×(2.0–) 3.5 µm±1.0 µm (–6.5) (l/w = (1.6–) 2.5±0.7 (–4.5), n = 29). Whorls consisted of (1–) 2–5 (–6) phialides.

#### Types

Holotype: Specimen CBS H-19247 at CBS (Germany: Niedersachsen; horseradish) (Figures 3b, 9g), an ex-holotype culture at CBS (CBS 124.64) included in this study as *V. longisporum* strain PD687 and submitted to NRRL (NRRL 54793), Stark [42] (p. 509) submitted permanent slides of type material to the Herbarium des Staatsinstitutes für Allgemeine Botanik Hamburg, these slides are missing at HBG.

#### Specimens examined

*Verticillium longisporum* strains PD348 (USA: CA; cauliflower), PD356 (USA: IL; horseradish) and PD687 (Germany: Niedersachsen; horseradish) (Table S1), representing the three lineages of *V. longisporum* [27], and the holotype specimen CBS H-19247 (Germany: Niedersachsen; horseradish), a dried agar culture (Figures 3b, 9g), were examined in this study.

#### Distribution and host range

Currently known from France, Germany, Japan, Sweden and USA (CA, IL). Substrates include birdrape, cabbage, cauliflower, horseradish, radish, rape, sugar beet and wild radish [27].

#### Commentary

*Verticillium longisporum* is a diploid hybrid that originated at least three different times from four different parental lineages in three different species, including *V. dahliae*, Species A1 and Species D1 (Figure 1) [27]. *Verticillium dahliae* is the only known parent of *V. longisporum*, Species A1 and Species D1 have never been collected [27]. The holotype of *V. longisporum* represented by ex-holotype strain PD687 belongs to *V. longisporum* lineage A1/D3 that is one of the three lineages of *V. longisporum*, and *V. longisporum* is thus polyphyletic [27]. There is general agreement that fungal species should be monophyletic. However, we decided that *V. longisporum* should remain a polyphyletic species, because it seems impractical to name each lineage of *V. longisporum*. We currently know of three lineages of *V. longisporum* that represent three independent hybridization events, but there might be many more. Little is known about fungal hybrids, but in plants, hybrids can evolve frequently over short periods in small areas [43].

We included the ex-holotype isolate *V. longisporum* strain PD687 in our studies, strain PD687 did not form any microsclerotia. But microsclerotia were present in the holotype that is a dried culture of strain PD687 (CBS 124.64) (Figure 3b). The microsclerotia in the holotype documented in Figure 9g were similar to the ones described by Stark [42] on page 500 for ‘Typ X’ as *V. longisporum* was referred to prior to its description. Thus, *V. longisporum* strain PD687 likely lost its ability to produce microsclerotia due to prolonged culturing.

Karapapa et al. [24] compared *V. longisporum* to the morphologically similar *V. dahliae*, and found that *V. longisporum* microsclerotia and conidia were longer than the ones in *V. dahliae*, and that *V. longisporum* conidiophores had fewer phialides in each whorl than *V. dahliae*.

We evaluated those characters and found that for the isolates used in this study grown on PDA, microsclerotia and conidia size might be useful to distinguish *V. longisporum* from *V. dahliae*. In *Verticillium longisporum* strain PD356, the majority of microsclerotia were elongate (Figure 9d), but rounded microsclerotia were still present (Figure 9e), and in some sectors of the colony, rounded microsclerotia were in the majority (Figure 9e). In *V. longisporum* strain PD348, there were roughly as many elongate microsclerotia as there were rounded microsclerotia. *Verticillium dahliae* microsclerotia were mostly rounded, but in some areas elongate microsclerotia were prevalent. The short brown-pigmented hyphae that were frequently attached to microsclerotia (Figure 9f) are possibly immature microsclerotia as illustrated by Isaac [22]. Similar structures were seen in this study in *V. dahliae* (Figure 6k). The third strain of *V. longisporum* investigated here, the ex-holotype strain PD687 did not form any microsclerotia. Conidia of *V. longisporum* were on average 8.5×3.5 µm (Figure 9c) and conidia of *V. dahliae* 6.5×3.0 µm (Figure 6h). However, conidia lengths might also at times be misleading, as the size ranges overlap, standard errors were 2.5 and 1.5 µm, respectively. We found that both *V. longisporum* and *V. dahliae* had similar numbers of phialides in each whorl, 2–4 for *V. dahliae*, and 2–5 for *V. longisporum*, this is unlike that proposed by Karapapa et al. [24] who reported 4–5 in *V. dahliae* and mostly 3 in *V. longisporum*. In our hands, *Verticillium longisporum* strain PD348 very frequently had 5 phialides per whorl.

Thus, a combination of conidia length and microsclerotia morphology might in many cases yield correct species identifications, but the two characters will also be misleading at times.

Another differentiating character was given by Stark [42]. He found that *V. longisporum* culture filtrate fluoresced, whereas fluorescence was absent in *V. dahliae*. We did not investigate fluorescence in the two species.

***Verticillium nonalfalfae*** **Inderb., H. W. Platt, R. M. Bostock, R. M. Davis & K. V. Subbarao,** ***sp. nov.*** **Figure 10**

MycoBank: MB563555

Etymology: Known to occur on a variety of hosts, but not *Medicago sativa* (‘alfalfa’).

#### Latin diagnosis

Verticillio alfalfae morphologia simile, sed characteribus sequentiarum nucleidearum distinguendum. Actin positione 16 (C), 63 (A); Elongation factor 1-alpha: positione 148 (C), 179 (G), 190 (C), 248 (G), 316 (G), 332 (G), 342 (T), 414 (G), 470 (T), 473 (C), 494 (G), 513 (T), 541 (C), 580 (G), 595 (T), 597 (C), 639 (T); Glyceraldehyde-3-phosphate dehydrogenase positione 234 (T), 267 (T); Tryptophan synthase positione 471 (C), 534 (C).

#### Description

Colonies on PDA after two weeks 3.5–5.5 cm, white at first, later darkening due to the formation of resting mycelium immersed in the agar (Figures 10a, 10b). Aerial mycelium generally abundant, floccose to pruinose, hyphae smooth-walled, 1.5–3 µm wide. Conidiophores erect or slanted (Figures 10c, 10d), generally determinate, branched or unbranched (Figure 10c, 10d), formed disjointedly throughout the colonies, hyaline, 30–710 µm in length, 4.5–6 µm wide, narrowing towards the apex to 2–3 µm, transversely septate, septa spaced more narrowly towards the apex. Conidiogenous cells are phialides (Figure 10e), arranged in (1–) 2–6 whorls along conidiophores (Figures 10d 10e). Whorls spaced 50–160 µm apart, closer towards the apex, consisting of (1–) 2–5 (–7) phialides (Figure 10e), arising below transverse septum. Apical whorls consisting of one apical and one to several lateral phialides (Figures 10d, 10e). Phialides subulate, tapering from 2–3 µm at the base to 1–1.5 µm at the tip, terminal phialides 40–60 µm long, lateral phialides 30–45 µm long (Figure 10e). Conidia hyaline, smooth-walled, cylindrical with rounded apices to oval (Figure 10f), allantoid at times, (4.0–) 6.0 µm±1.0 µm (–10.5)×(2.5–) 3.0 µm±0.5 µm (–3.5) (l/w = (1.3–) 2.0±0.2 (–2.7), n = 80), accumulating at the tip of the phialides (Figure 10d). Resting mycelium present (Figures 10g, 10h, 10i), consisting of brown-pigmented hyphae, up to 9 µm wide, thick-walled, straight or curved, solitary or aggregated (Figures 10g, 10h), torulose at times (Figure 10i).

#### Types

Holotype: Dried culture of *V. nonalfalfae* strain PD592 (Japan: Hokkaidou; Irish potato) deposited at UC (UC 1953898), an ex-holotype culture at CBS (CBS 130339) and NRRL (NRRL 54791).

#### Specimens examined

The description was based on *V. nonalfalfae* strains PD592 (Japan: Hokkaidou; Irish potato), PD616 and PD626 (UK; common hop), PD744 (Cuba; potato field soil), PD745 (Canada: Manitoba; spinach), PD808, PD809 and PD811 (Slovenia; common hop) and PD810 (Slovenia: petunia) (Table S1).

#### Distribution and host range

Currently known from Canada, Cuba, Japan, Slovenia and UK. Substrates include common hop, Irish potato, petunia and spinach.

#### Commentary

*Verticillium nonalfalfae* is morphologically indistinguishable from *V. alfalfae*, but the two species differ in pathogenicity. *Verticillium nonalfalfae* causes disease on a variety of different hosts, whereas *V. alfalfae* causes disease mainly on lucerne [44]. Other differences include vegetative compatibility groups [45], mating types (Figure 2), as well as the DNA characters listed in the species descriptions. *Verticillium alfalfae* and *V. nonalfalfae* were described as new species because no synonyms of the morphologically similar *V. albo-atrum* were available (www.indexfungorum.org, accessed on September 30, 2011).

*Verticillium nonalfalfae* and *V. alfalfae* have long been recognized as two genetically distinct groups referred to as non-lucerne and lucerne pathotype, respectively [46], [47], [48].

Within *Verticillium*, *V. nonalfalfae* and *V. alfalfae* lack unique, diagnostic morphological characters and were frequently confused with the distantly related *V. albo-atrum*. The three fungi share an overall similar morphology, including the formation of resting mycelium (Figures 4g, 4h, 5f, 5g, 5h, 10g, 10h, 10i). *Verticillium albo-atrum* also forms microsclerotia (Figures 4i, 4j, 4k), one-septate, brown-pigmented conidia (Figure 4f), as well as phialides that originate directly from conidia (Figure 4f). However, microsclerotia were only observed on WA-p and on PLYA media, not on PDA medium, and one-septate, brown-pigmented conidia, and conidia germinating by phialide formation are relatively rare. Thus, based on our data, it is not possible to consistently differentiate *V. nonalfalfae* and *V. alfalfae* from *V. albo-atrum* using morphological characters. *Verticillium albo-atrum* may co-occur with *V. nonalfalfae* on some hosts, as Keyworth [49] isolated *Verticillium* strains forming resting mycelium, as well as *Verticillium* strains forming microsclerotia and resting mycelium simultaneously, from diseased potato plants in Connecticut.

***Verticillium nubilum*** **Pethybr., Transactions of the British Mycological Society 6: 117 (1919)** **Figure 11**

MycoBank: MB225664

#### Description

Colonies on PDA after two weeks 2.5–6 cm diam, white at first, later darkening due to the chlamydospores immersed in the agar (Figures 11a, 11b). Aerial mycelium generally abundant, floccose to pruinose, hyphae smooth-walled, (1–) 2–4 µm wide. Conidiophores present (Figure 11c). Conidiogenous cells are phialides (Figure 11d) arranged in whorls along conidiophores (Figure 11c), arising below transverse septum. Whorls consisting of one or more phialides (Figures 11c, 11d). Phialides subulate (Figure 11d). Conidia hyaline, smooth-walled, cylindrical with rounded apices to oval (Figure 11e), allantoid at times, rarely with central septum, (4.5–) 7.5 µm±2.0– µm (–14.5)×(2.0–) 2.5 µm±0.5 µm (–3.5) (l/w = (2.0–) 3.0±0.5 (–5.0), n = 50) (Figure 11e). Chlamydospores present, rounded to elongate, 6–14 µm diam, solitary or in chains of up to 6, straight or curved (Figures 11f, 11g, 11h). Brown-pigmented hyphae present at times (Figures 11h, 11i), generally attached to chlamydospores (Figure 11h).

#### Types

Holotype: Missing, not at DBN, IMI, K; Lectotype (designated herein): Illustration from protolog: Figure 5 on Plate 4 in Pethybridge [50], available online from Cyberliber, an Electronic Library for Mycology at http://www.cybertruffle.org.uk/cyberliber/59351/0006/002/p004b.jpg (accessed on October 5, 2011); Epitype (designated herein): Dried culture of *Verticillium nubilum* strain PD742 (obtained from CBS as CBS 457.51)(UK; soil) deposited at UC (UC 1953894) and NRRL (NRRL 54796).

#### Specimens examined

The description was based on *V. nubilum* strains PD621 (UK; mushroom compost), PD702 (UK; Irish potato), PD741 (UK; soil), PD742 (UK; soil) (Table 1).

#### Distribution and host range

Currently known from the UK. Substrates include mushroom compost, Irish potato and soil.

#### Commentary

*Verticillium nubilum* was described by Pethybridge [50] from the surface of a potato tuber attacked by *Phytophthora infestans*. The protolog of *V. nubilum* contains descriptions of the *V. nubilum* morphology and a photograph of chlamydospores, but no reference is made to type material. We inquired at Kew (K), CABI Bioscience (IMI) and Dublin (DBN), none of which has any *V. nubilum* type material in its possession. Isaac [23] who studied *V. nubilum* in detail did not mention any herbarium material. We did not find any *V. nubilum* cultures by Pethybridge in any of the major culture collections (CBS, IMI, DSMZ, ATCC). Thus, in absence of any original fungal material, we designated the illustration from the *V. nubilum* protolog, Figure 5 on Plate 4 in Pethybridge [50], as the lectotype for *V. nubilum*.

Isaac [23] studied *V. nubilum* in detail and submitted several strains to CBS, of which we selected a dried culture of strain PD742 (CBS 457.51) as epitype. Our observations of *V. nubilum* agreed with the accounts by Pethybridge [50] and Isaac [23]. Pethybridge [50] noted that *V. nubilum* conidia were larger than those of *V. albo-atrum*. We found that *V. nubilum* condia were on average 7.5×2.5 µm (Figure 11c), the largest in *Verticillium*, with the exception of *V. longisporum* conidia that were on average 8.5×3.5 µm (Figure 9c). Differing from both Pethybridge [50] and Isaac [23], small numbers of brown-pigmented hyphae not directly associated with chlamydospores were sometimes present (Figure 11i), but these were lighter colored than the resting mycelium in other species (eg Figures 4g, 4h).

All the *V. nubilum* isolates that we examined formed very few conidia and conidiophores, which prevented us from conclusively assessing conidiophore morphology and dimensions. However, the few conidiophores and phialides that we saw were similar to other *Verticillium* species, in both morphology and dimensions (Figures 11c, 11d). *Verticillium nubilum* can be differentiated from other *Verticillium* species by the near exclusive formation of chlamydospores as resting structure (Figures 11f, 11g, 11h), in combination with the relatively large conidia (Figure 11c), but can be confused with *Gibellulopsis nigrescens* that forms distinctly smaller chlamydospores [15], [23].

***Verticillium tricorpus*** **I. Isaac, Transactions of the British Mycological Society 36(3): 194 (1953)** **Figures 3c** **,** **12**

**Figure 12:**
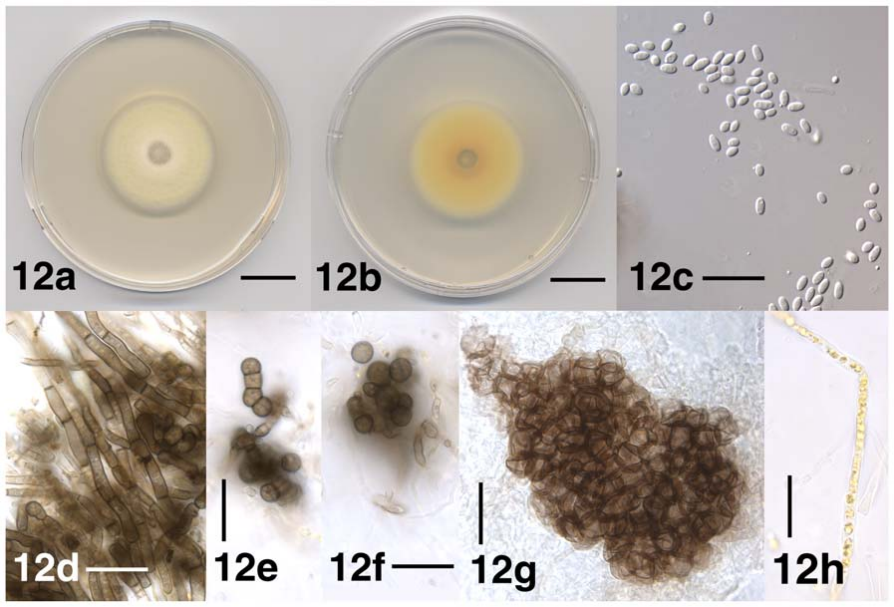
Select morphological features of *Verticillium tricorpus*. 12a. Colony of strain P685 after 10 days on PDA, frontal view. 12b. Colony of strain PD685 after 10 days on PDA, reverse view. 12c. Conidia of strain PD685 after 38 days on PDA. 12d. Resting mycelium of strain PD685 after 38 days on PDA. 12e. Chain of chlamydospores and microsclerotium of strain PD685 after 38 days on PDA. 12f. Microsclerotium of strain PD685 after 38 days on PDA. 12g. Microsclerotium of lectotype specimen IMI 51602. 12h. Yellow-pigmented hypha of strain PD685 after 38 days on PDA. Scale bar: 12a, 12b = 1 cm; 12c–12h = 20 µm; Imaging method: 12a, 12b = DS; 12c = DIC; 12d–12h = BF.

MycoBank: MB307745

#### Description

*Verticillium tricorpus* was described in detail by Isaac [23]. We provide illustrations of the culture morphology (Figures 12a, 12b), the conidia (Figure 12c), resting structures including resting mycelium, chlamydospores and microsclerotia (Figures 12d, 12e, 12f, 12g), and yellow-pigmented hyphae (Figure 12h).

#### Types

Holotype: Missing, not found at K, IMI, CBS, an ex-holotype culture (UK; garden tomato) available (IMI 51602, CBS 447.54), culture CBS 447.54 included in this study as *V. tricorpus* strain PD690 and submitted to NRRL (NRRL 54794); Lectotype (designated herein): Specimen K(M) 172015, originally IMI 51602 (England: Fareham, South Hampshire; wilted garden tomato), marked ‘isotype ?’ (Figures 3c, 12g).

#### Specimens examined

*Verticillium tricorpus* strains PD593 (Japan; Irish potato), PD594 (Japan: Chiba; garden tomato), PD685 (Japan; larkspur), PD690 (UK; garden tomato), and PD703 (Netherlands; carnation) (Table S1), as well as *V. tricorpus* lectotype specimen IMI 51602 (UK; garden tomato) were included in this study (Figures 3c, 12g).

#### Distribution and host range

Currently known from Japan, Netherlands and UK. Substrates include carnation, garden tomato, Irish potato and larkspur.

#### Commentary

Isaac [23] (p. 194) deposited *V. tricorpus* type material at IMI, K and CBS. We were only able to locate specimen IMI 51602, a dried *V. tricorpus* culture on PDA medium labeled ‘isotype ?’ (Figure 3c). Specimen IMI 51602 is likely derived from ex-type strain IMI 51602 deposited at CBS by I. Isaac as strain CBS 447.54. Thus, since the holotype appeared to be missing, we designated specimen IMI 51602, a likely isotype, as the lectotype of *V. tricorpus* (Art. 9.2, Art. 9.9, Art. 9.10). Specimen IMI 51602 did not display typical *V. tricorpus* morphology. Whereas verticillate conidiophores and microsclerotia were present (Figure 12g) in agreement with the description provided by Isaac [23], yellow-pigmented hyphae, chlamydospores and resting mycelium were absent. However, according to the ICBN, lectotypes have to be chosen from among isotypes if they exist (Art. 9.10). *Verticillium tricorpus* specimen IMI 51602 is a likely isotype and was thus designated as lectotype. Upon initial culturing, *Verticillium tricorpus* colonies on agar medium are yellow to orange (Figures 12a, 12b) due to the presence of yellow-pigmented hyphae (Figure 12h). Resting mycelium, chlamydospores and microsclerotia are also formed simultaneously (Figures 12d, 12e, 12f, 12g). The yellow to orange coloration is typically less intense after prolonged culturing, or if obscured by resting structures. *Verticillium tricorpus* is morphologically indistinguishable from *V. isaacii* and *V. klebahnii*. All three species are characterized by the formation of resting mycelium (Figures 7f, 8e, 12d), chlamydospores (Figures 7g, 8f, 12e) and microsclerotia (Figures 7h, 8h, 12f, 12g), as well as yellow-pigmented hyphae (Figure 7i, 8h, 12h) that confer agar cultures yellow to orange coloration (Figure 7b, 8b, 12b).

There is evidence for differences in pathogenicity. Whereas *V. isaacii strains* PD343, PD610–PD613 were not pathogenic on lettuce or artichoke [41], *V. klebahnii* strain PD401 was pathogenic on lettuce [51]. *Verticillium tricorpus* is only pathogenic on tomato [23].

**Figure 13:**
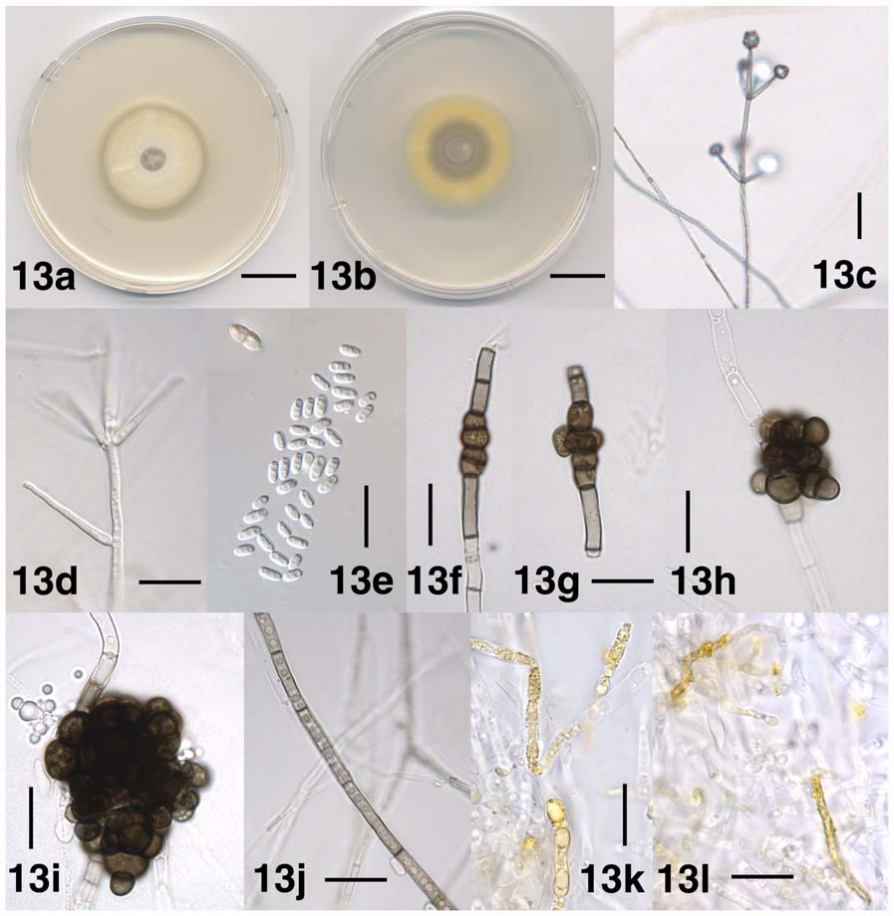
Morphological features of *Verticillium zaregamsianum*. 13a. Colony of strain PD736 after 10 days on PDA, frontal view. 13b. Colony of strain PD736 after 10 days on PDA, reverse view. 13c. Conidiophore of strain PD736 after 32 days on WA-p. 13d. Solitary phialide of strain PD733 after 31 days on WA-p. 13e. Conidia of strain PD736 after 44 days on PDA; Inset: Brown, septate and constricted conidium of strain PD733 after 44 days on WA-p. 13f–13i. Microsclerotia of strain PD586 after 31 days on WA-p. 13f. Microsclerotium initial resembling chlamydospores. 13g. Microsclerotium initial with cells that originated by lateral cell divisions. 13h. Small microsclerotium. 13i. Microsclerotium. 13j. Hypha of resting mycelium in strain PD586 after 31 days on WA-p. 13k. Hyphal cells of strain PD733 containing yellow pigment after 44 days on PDA. 13l. Hyphal cells of strain PD736 encrusted by yellow crystals after 10 days on PDA. Scale bar: 13a, 13b = 2 cm; 13c = 50 µm; 13d–13l = 20 µm. Imaging method: 13a, 13b = DS; 13c, 13f–13l = BF; 13d, 13e = DIC.

***Verticillium zaregamsianum*** **Inderb., T. Usami, Takeshi Kanto, R. M. Bostock, R. M. Davis & K. V. Subbarao,** ***sp. nov.*** **Figure 13**

MycoBank: MB563556

Etymology: Named after Rasoul Zare and Walter Gams who collaboratively established the modern taxonomic framework this study is based on.

#### Latin diagnosis

Verticillio dahliae simile sed pigmentum croceum exsudans.

#### Description

Colonies on PDA after two weeks 3–6.5 cm, white at first, later yellow, reverse orange to yellow, then darkening due to the formation of microsclerotia (Figures 13a, 13b). Aerial mycelium generally abundant, floccose, hyphae smooth-walled, 1–4 µm wide. Conidiophores erect or slanted (Figures 13c, 13d), generally determinate, branched or unbranched, formed disjointedly throughout the colonies, hyaline, 50–800 µm in length, 3–4 µm wide, narrowing towards the apex to 2–3 µm, transversely septate, septa spaced more narrowly towards the apex. Conidiogenous cells are phialides (Figure 13d), arranged in (1–) 3–7 (–11) whorls along conidiophores (Figures 13c, 13d). Whorls spaced 25–100 µm apart, closer towards the apex, consisting of (1–) 2–5 (–6) phialides, arising below transverse septum. Apical whorls consisting of one apical and one to several lateral phialides (Figures 13c, 13d). Phialides subulate, tapering from 2–3 µm at the base to 1–1.5 µm at the tip, terminal phialides 25–60 µm long, lateral phialides 20–60 µm long (Figure 13d). Conidia hyaline, smooth-walled (Figure 13e), cylindrical with rounded apices to ellipsoidal, (4.0–) 5.5 µm±1.0 µm (–12.5)×(2.0–) 3.0 µm±0.5 µm (–6.5) (l/w = (1.4–) 2.0±0.3 (–2.8), n = 88), accumulating at the tip of the phialides (Figure 13c), one-septate, constricted at septum, and brown-pigmented at times with age (Figure 13e). Microsclerotia regularly or irregularly distributed throughout the colony, rounded to variously shaped, up to 90 µm diam and consisting of rounded cells, up to 14 µm diam (Figures 13f, 13g, 13h, 13i). Structures resembling chlamydospores, possibly microsclerotia initials, present at times, up to 10 µm wide (Figures 13f, 13g). Scattered brown-pigmented hyphae present at times, thick-walled, up to 5 µm wide (Figure 13j). Yellow-pigmented hyphal cells present (Figures 13k, 13l), up to 6 µm wide, containing globules of yellow pigment (Figure 13k), at times yellow pigmented crystals present outside the cells (Figure 13l).

#### Types

Holotype: Dried culture of *V. zaregamsianum* strain PD736 (Japan: Chiba; lettuce) deposited at UC (UC 1953899), an ex-holotype culture at CBS (CBS 130342) and NRRL (NRRL 54795).

#### Specimens examined

The description was based on *V. zaregamsianum* strains PD586, PD739 and PD740 (Japan: Chiba; tenweeks stock), PD731, PD733 and PD734 (Japan: Hyogo; lettuce), PD735 (Japan: Kagawa; lettuce), PD736, PD737 and PD738 (Japan: Chiba; lettuce) (Table S1).

#### Distribution and host range

Currently only known from Japan. Substrates include lettuce and tenweeks stock.

#### Commentary

*Verticillium zaregamsianum* differs from all other *Verticillium* species by the formation of microsclerotia (Figures 13h, 13i) simultaneously with yellow-pigmented hyphae (Figures 13k, 13l). Only a few potential chlamydospores, possibly immature microsclerotia (Figure 13f, 13g), and sparse resting mycelium (Figure 13j) were observed. *Verticillium tricorpus*, *V. isaacii* and *V. klebahnii* differ by the formation of abundant chlamydospores (Figures 7g, 8f, 12e) and resting mycelium (Figures 7f, 8f, 12d). *Verticillium zaregamsianum* was described as a new species because according to Index Fungorum (www.indexfungorum, accessed on September 30, 2011), there were no synonyms available for *V. tricorpus*. None of the two synonyms of the morphologically similar *V. dahliae* listed in Index Fungorum (*V. ovatum* G.H. Berkeley & A.B. Jackson, *V. tracheiphilum* Curzi) matched the morphology of *V. zaregamsianum* in that no yellow-pigmented hyphae were mentioned [52], [53].

## Discussion

We have generated a solid taxonomic framework for *Verticillium* that recognizes ten species, five of which are new to science. Our results show that resting structure morphology, traditionally the most important morphological character to differentiate *Verticillium* species still plays a part in species identification, but the near-complete reliance on resting structure morphology to identify *Verticillium* species will have to be abandoned.

As other recent studies of fungal diversity [54], [55], [56], [57], [58], [59], [60], our approach combined phylogenetic analyses, literature research and morphological comparisons, and established that each *Verticillium* species, except the hybrid *V. longisporum*, corresponded to a single group in the phylogenetic tree. We included ex-type strains that are derived from herbarium type material to which fungal names are permanently linked according to the International Code of Botanical Nomenclature (ICBN). All species-level phylogenetic groups contained a single ex-type strain that thus conferred a species name to all current and future group members guaranteeing taxonomic stability.

This study recognized all previously known species of *Verticillium* [15]. These were *V. albo-atrum* [21], *V. dahliae* [35], *V. longisporum* [24], *V. nubilum* [50] and *V. tricorpus* [23]. In order to stabilize the application of names, we selected several new types. For *V. albo-atrum* and *V. nubilum*, we designated illustrations as lectotypes since no herbarium material was available, and for *V. tricorpus* an isotype was designated as lectotype. For *V. dahliae* and *V. albo-atrum*, epitypes were selected based on our morphological comparisons, and a *V. nubilum* epitype was chosen among strains deposited by Isaac [20], who studied *V. nubilum* in detail.

### The five new *Verticillium* species

Five species-level phylogenetic groups did not contain any ex-type strains, and were thus described as new species (Figure 1). These were *V. alfalfae* (Figure 5) and *V. nonalfalfae* (Figure 10) that are relatives of *V. dahliae* (Figure 6) and *V. longisporum* (Figure 9), as well as *V. zaregamsianum* (Figure 13), *V. isaacii* (Figure 7) and *V. klebahnii* (Figure 8), all related to *V. tricorpus* (Figure 12).

The sister species *Verticillium alfalfae* and *V. nonalfalfae* (Figure 1) were previously referred to as the respective lucerne and non-lucerne pathotypes of *‘V. albo-atrum’* [46], and have long been recognized as two genetically distinct groups [47], [48]. The two species are morphologically indistinguishable, but differ in pathogenicity. *Verticillium nonalfalfae* causes disease on a variety of hosts whereas *V. alfalfae* causes disease on lucerne [44]. Other differences include vegetative compatibility groups [45], mating types (Figure 2), as well as the DNA characters listed in the species descriptions. Molecular data have previously been included in species descriptions [55], [58], [59], [61], [62]. We did not detect any genetic variation within *V. alfalfae* and *V. nonalfalfae* (Figure 1). However, variation within *V. nonalfalfae* has been demonstrated using AFLP markers and a proteomics approach [63], [64], [65], [66].

*Verticillium alfalfae* and *V. nonalfalfae* are related to *V. dahliae* and *V. longisporum* (Figure 1), but differ morphologically by the formation of resting mycelium. However, resting mycelium is also present in the distantly related *V. albo-atrum* with which *V. alfalfae* and *V. nonalfalfae* have frequently been confused [15].

The existence of two distantly related groups in *Verticillium* forming resting mycelium was established earlier. *Verticillium alfalfae* and *V. nonalfalfae* have been referred to as ‘*V. albo-atrum*’ group 1, and *V. albo-atrum* as ‘*V. albo-atrum*’ group 2 [67], [68]. Robb et al. [67] suggested that ‘*V. albo-atrum*’ group 2 was characterized by the formation of brown-pigmented hyphae aggregating in bundles, whereas brown-pigmented hyphae in ‘*V. albo-atrum*’ group 1 were solitary. However, we found bundles of brown-pigmented hyphae in both *V. alfalfae* (Figure 5g), *V. nonalfalfae* and *V. albo-atrum* (Figure 4h), and thus, this character is not suitable for species differentiation.

It was earlier suggested that ‘*V. albo-atrum*’ groups 1 and 2 may constitute different species [68], [69], but details have been unclear. Using comparisons to the *V. albo-atrum* type description and illustrations [21], it was apparent that *V. albo-atrum* corresponded morphologically to ‘*V. albo-atrum*’ group 2. *Verticillium albo-atrum* forms microsclerotia (Figures 4i, 4j, 4k) in addition to resting mycelium [69], [70], [71] (Figures 4g, 4h), as well as yellow-pigmented hyphae as observed by Klebahn [35] (Figures 4b, 4l). Recently, the name ‘*V. albo-atrum*’ has possibly been applied more frequently to ‘*V. albo-atrum*’ group 1 now comprising *V. alfalfae* and *V. nonalfalfae*, than to ‘*V. albo-atrum*’ group 2, now *V. albo-atrum*. Thus, in the absence of molecular data, detailed morphological descriptions or cultures, it is not possible to relate the previous literature on ‘*V. albo-atrum*’ with absolute certainty to the current species concepts of *V. albo-atrum*, *V. alfalfae* and *V. nonalfalfae*. Adding to the confusion, there might be additional groups with similar morphology, as in a study of ‘*V. albo-atrum*’ diversity isolates from pea formed a separate cluster [46], [68].

The remaining three new species proposed here, *V. isaacii*, *V. klebahnii* and *V. zaregamsianum* are related to *V. tricorpus* (Figure 1). *Verticillium isaacii*, *V. klebahnii* and *V. tricorpus* are morphologically indistinguishable, they are characterized by the formation of resting mycelium (Figures 7f, 8e, 12d), chlamydospores (Figures 7g, 8f, 12e) and microsclerotia (Figures 7h, 8h, 12f), as well as the presence of yellow-pigmented hyphae (Figure 7i, 8h, 12h), providing the colonies on agar medium with a yellow or orange coloration (Figure 7b, 8b, 12b). The three species are a monophyletic group (Figure 1), and they could have been considered as three different lineages within just one species, *V. tricorpus*. However, compared with other *Verticillium* species, *V. tricorpus*, including what are now *V. isaacii* and *V. klebahnii*, was known to be very diverse, both in terms of ITS sequence data [48] and vegetative compatibility groups [72]. There is evidence for differences in pathogenicity. *Verticillium tricorpus* was only pathogenic on tomato [23], *V. klebahnii* was pathogenic on lettuce [51], and *V. isaacii* was not pathogenic on either lettuce or artichoke [41]. Further research is needed to determine the host ranges of these species.

*Verticillium zaregamsianum*, the third new species related to *V. tricorpus* (Figure 1), is morphologically distinct from all other *Verticillium* species, *V. zaregamsianum* forms predominantly microsclerotia (Figure 13i), as well as yellow-pigmented hyphae (Figures 13k, 13l). *Verticillium zaregamsianum* is a pathogen of lettuce in Japan [73].

Table 2 provides an overview of the taxonomic changes made in this paper and relates the new taxonomic system to previously described species.

**Table 2:** Correspondence of previous to current taxonomic system and summary of taxonomic changes enacted.

Previous names	Taxonomic changes	Current names
*Verticillium albo-atrum*	Split into three species, designation of epitype for *V. albo-atrum*	a *V. albo-atrum*, *V. alfalfae* or *V. nonalfalfae*
*Verticillium dahliae*	Epitype specimen designated	*V. dahliae*
*Verticillium longisporum*	None	*V. longisporum*
*Verticillium nubilum*	Epitype specimen designated	*V. nubilum*
*Verticillium tricorpus*	Split into three species, designation of lectotype for *V. tricorpus*	*V. tricorpus*, *V. isaacii* or *V. klebahnii*
-	Described as new species	b *V. zaregamsianum*

a *V. albo-atrum* is more closely related to *V. tricorpus* than to *V. dahliae*, whereas *V. alfalfae* and *V. nonalfalfae* are closely related to *V. dahliae* (Figure 1).

b *V. zaregamsianum* was referred to as *V. tricorpus* at least once [73], but differs morphologically.

### Phylogenetic relationships of *Verticillium* species

In agreement with previous studies [15], [74], we identified two major groups in *Verticillium* that we named Clades Flavexudans and Flaxnonexudans, respectively (Figure 1). Clade Flavexudans comprised all species that produced yellow-pigmented hyphae that were absent in all members of Clade Flavnonexudans. Whereas Clade Flavexudans was well supported by the phylogenetic analyses, Clade Flavnonexudans, in particular the monophyly of *V. nubilum* with the remaining members of Clade Flavnonexudans, only received support in the parsimony analyses (Figure 1). More research is needed to conclusively determine the phylogenetic placement of *V. nubilum* within *Verticillium*.

The phylogenetic relationships within the major clades were well resolved (Figure 1). Within Clade Flavexudans, the branching order of *V. albo-atrum*, *V. isaacii*, *V. klebahnii*, *V. tricorpus* and *V. zaregamsianum* had maximal support in all analyses. The topology of Clade Flavnonexudans was also well resolved, except for the placement of Species A1, an ancestor of the diploid hybrid *V. longisporum*. Species A1 that is unknown and has never been collected [27], is basal to the clade of *V. alfalfae*, *V. dahliae*, *V. nonalfalfae* as well as Species D1, another unknown species and second ancestor of *V. longisporum* [27], but only supported by the Bayesian analyses (Figure 1). Inderbitzin et al. [27] studied the evolutionary history of *V. longisporum* in detail, they found that *V. longisporum* evolved at least three different times from four different parental lineages representing three different species. The results of Inderbitzin et al. [27] differ from the current study with regard to the placement of Species A1 that formed a clade with Species D1 and *V. dahliae*, whereas in this study, Species A1 was a sister group to the clade of *V. alfalfae*, *V. dahliae*, *V. nonalfalfae* and Species D1. The topological divergence involving Species A1 might be due to differences in taxon sampling and the use of an additional locus for phylogenetic analyses in Inderbitzin et al. [27].

### Hosts and geographic distribution of *Verticillium* species

The isolates used in this study represent only a small fraction of the vast literature on *Verticillium* [1] and therefore do not paint a complete picture on geographic distribution and host range. However, the data provided here and in Inderbitzin et al. [27] are associated with correctly identified isolates and constitute an initial approximation of the distributions and host associations of *Verticillium*. For *V. dahliae*, *V. longisporum* and *V. nubilum* distribution and host range data are in general agreement with the literature [1], [23], [50], [75]. *Verticillium dahliae* is known from four continents and fourteen host families, and is by far the most widespread *Verticillium* species. This contrasts with *V. nubilum* that is only known from Irish potato in the UK, and with *V. longisporum* that occurs in Europe, Japan and North America but is restricted mainly to hosts in the Brassicaceae. More work is needed to expand our knowledge on the distributions and host ranges of the remaining species, including the newly described *V. alfalfae*, *V. isaacii*, *V. klebahnii*, *V. nonalfalfae* and *V. zaregamsianum*, as well as *V. albo-atrum* and *V. tricorpus* that are now more narrowly defined.

### Identification of *Verticillium* species

Correct and consistent identification is crucial for effective and efficient disease control [76], but we found *Verticillium* species may frequently have been misidentified. Based on DNA sequencing and phylogenetic analyses, we determined that at least 34 of the 293 isolates used in this study and the study by Inderbitzin et al. [27], were not correctly identified (Table 3). Given that the majority of *Verticillium* strains were from *Verticillium* research labs, the error rate among non-specialists is likely to be higher.

**Table 3:** Names of misidentified isolates received are given in top row, approximate correct names based on DNA sequencing and comparison to GenBank are in left column, numerals refer to numbers of isolates in each category.

Incorrect name^a^/Correct name	*V. albo-atrum*	*V. dahliae*	*V. longisporum*	*V. nubilum*	*V. tricorpus*
*V. albo-atrum*	-	1	-	-	-
*V. dahliae*	-	-	1	-	-
*V. tricorpus*	1	5	-	-	-
*Gibellulopsis nigrescens*	5	-	-	-	-
*Leptodontidium* sp.	-	-	-	1	-
*Lecanicillium* sp.	3	-	-	-	-
*Musicillium theobromae*	-	-	-	-	1
*Nectria* sp.	1	10	-	-	-
*Neosartorya* sp.	-	1	-	-	-
*Plectosphaerella* sp.	-	4	-	-	-

a A total of 34 incorrectly identified isolates were among the 293 isolates from this study and Inderbitzin et al. [27]. Origins of misidentified isolates available upon request.

*Verticillium* is difficult to separate from similar genera as it lacks morphological characters that are unique. The most conspicuous characters of *Verticillium*, the conidiophores bearing whorls of conidiogenous cells, as well as the resting structures, are also present in other genera including *Gibellulopsis* and *Musicillium*. This problem is illustrated by the fact that 26 of the 34 misidentified isolates belonged to genera other than *Verticillium* (Table 3).

Within *Verticillium*, resting structure morphology, conidia size, conidiophore size and pigmentation, the number of phialides per whorl, and the formation of yellow-pigmented hyphae has been used to differentiate species [23], [24], [35], [50]. It is known that resting structure morphology may vary depending on culture medium [50], [77] and other environmental conditions [78], and that yellow-pigmented hyphae may be lost after prolonged culturing [23]. We did not investigate the influence of environmental conditions on *Verticillium* morphology in detail, but found that differences in resting structures between *V. albo-atrum*, *V. alfalfae* and *V. nonalfalfae* were more readily observed on WA-p and PLYA media than on PDA medium. However, the yellow coloration conferred to the agar medium by species in the Clade Flavexudans (Figure 1) was most prominent on PDA. Thus, based on our results, we recommend the combined use of PDA and WA-p for species identification.

**Figure 14:**
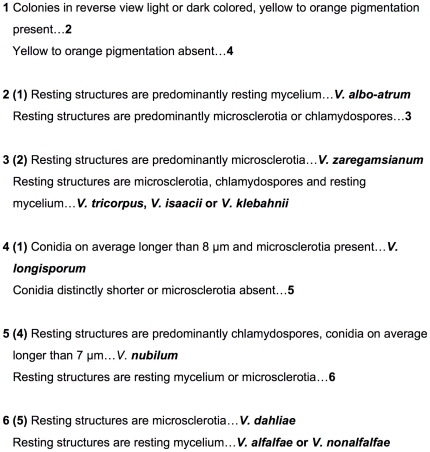
Key for the identification of *Verticillium* species from PDA medium using morphological characters.

In Figure 14 we provide a key to *Verticillium* species based on morphological characters. However, given the morphological variability of *Verticillium* species as discussed above, the key is more intended as an overview of *Verticillium* morphology than as an authoritative means for species identification. All results obtained using the key should be confirmed by DNA sequencing and phylogenetic analyses with ex-type isolates.

### Conclusions

The new taxonomic system presented here is based on a multifaceted approach that included phylogenetic and morphological investigations, herbarium and literature research, and allows for a more reliable and consistent identification of *Verticillium* species. We envision that over time, this taxonomic system will lead to a significant improvement of our knowledge of *Verticillium* biology. Potential practical applications are many, and may include more efficient and effective disease management strategies and quarantine regulations.

### Future Research

Future research will focus on the determination of host ranges of some of the new species of *Verticillium* as well as *V. albo-atrum*. Also, inclusion of more isolates from non-agricultural systems in studies of *Verticillium* diversity would be desirable.

## Materials and Methods

### Taxon selection, origin of fungal strains and DNA sequences retrieved from GenBank

Taxa were selected to cover the known diversity of *Verticillium* [46], and included 74 strains representing *V. tricorpus*, *V. nubilum*, *V. albo-atrum*, *V. longisporum* and *V. dahliae* as well as the outgroup *Gibellulopsis nigrescens* based on results from Zare et al. [15]. We previously clarified the phylogenetic relationship of *V. dahliae* and *V. longisporum* [27], and for *V. dahliae* and *V. longisporum* only included six taxa representing the main lineages of the two species. The isolates were obtained from a variety of different sources (Table S1), and initially identified based on morphology. Common names used for hosts were obtained from www.ITIS.gov accessed on June 6, 2010.

For the following 13 isolates DNA sequence data by Inderbitzin et al. [27] was retrieved from GenBank. *V. dahliae* strains PD322 (HQ206718, HQ206921, HQ414624, HQ414719, HQ414909) PD327 (HQ206723, HQ206925, HQ414628, HQ414723, HQ414913), PD502 (HQ206813, HQ206942, HQ414645, HQ414740, HQ414930); *V. alfalfae* strains PD338 (HQ206733), PD353 (HQ206742, HQ206933, HQ414636, HQ414731, HQ414921), PD620 (HQ206851, HQ206965, HQ414668, HQ414763, HQ414953), PD681 (HQ206891), PD682 (HQ206892); *V. nubilum* strain PD621 (HQ206852, HQ206966, HQ414669, HQ414764, HQ414954); *V. isaacii* strain PD660 (HQ206873, HQ206985, HQ414688, HQ414783, HQ414973); *V. longisporum* strains PD348 (HQ206738, HQ206930, HQ206931, HQ414633, HQ414634, HQ414728, HQ414729, HQ414918, HQ414919), PD356 (HQ206745, HQ206934, HQ206935, HQ414637, HQ414638, HQ414732, HQ414733, HQ414922, HQ414923), PD687 (HQ206893, HQ206993, HQ206994, HQ414696, HQ414697, HQ414791, HQ414792, HQ414981, HQ414982).

### Stock culture maintenance and growth conditions

All strains were single-conidium purified, and maintained as conidia suspensions at −80°C in glycerol diluted by half strength potato dextrose broth (25% glycerol vol/vol), and retrieved anew for each experiment. Cultures were grown on the following media. Potato dextrose agar (PDA) (Becton, Dickinson and Company, Sparks, MD), water agar (20 g agar/liter) (Becton, Dickinson and Company, Sparks, MD) supplemented with autoclaved stems of unidentified herbaceous plants in the Asteraceae and the Malvaceae (WA-p), and prune lactose yeast agar (PLYA) using food grade prune juice [79], [80]. Plates were sealed with Parafilm, and left on a lab bench inside a plastic container (crisper) subject to natural and artificial light and darkness at night. To document culture morphology, plates were left unsealed.

### Species recognition, description and naming

Species were defined as terminal or subterminal clades inferred from multigene phylogenetic analyses in accordance with the Genealogical Concordance Phylogenetic Species Recognition approach outlined by Taylor et al. [31] and named by the inclusion of ex-type strains. Except for the diploid hybrid *V. longisporum*, each species-level clade contained a single ex-type strain. New species were described for all species-level clades for which no existing names were available. Existing, readily available names include synonyms that are listed in Index Fungorum (www.indexfungorum.org). We were unable to search for additional synonyms among the 266 described *Verticillium* species listed in Index Fungorum (accessed September 30, 2011).

Morphological descriptions were based on cultures grown on PDA, WA-p and PLYA media. Microscopy was performed using a Leica DM5000 B microscope (Leica Microsystems CMS GmBH, Wetzlar, Germany), with bright field (BF), differential interference contrast (DIC) and phase contrast (PC) illumination of specimens mounted in water. Photographs were taken with a Leica DFC310 FX camera, using Leica Application Suite Version 3.6.0 software. Culture photographs were generated with a desktop document scanner (DS). The terminology used in the species diagnoses follows Kirk et al. [36]. For conidia dimensions, standard deviations are given. Nucleotide substitutions in the species diagnoses included all derived substitutions shared by all members of a species, except the substitutions that were in alignment regions of low complexity (single or multi-nucleotide repeats) or near gaps in regions of ambiguous alignment.

### Nomenclature

The electronic version of this document in itself does not represent a published work according to ICBN, and hence the new names contained in the electronic version are not effectively published under that Code from the electronic edition alone. Therefore, a separate edition of this document was produced by a method that assures numerous identical printed copies, and those copies were simultaneously distributed (on the publication date noted on the first page of this article) for the purpose of providing a public and permanent scientific record, in accordance with Article 29 of the Code. Copies of the print-only edition of this article were distributed on the publication date to botanical or generally accessible libraries of the following institutions, BPI, CBS, CUP, DAOM, HMAS, IMI, IRAN, NY, SFSU, TNS, UBC, and UC. The separate print-only edition is available on request from PLoS (Public Library of Science) by sending a request to PLoS ONE, Public Library of Science, 1160 Battery Street, Koshland Building East, Suite 100, San Francisco, CA 94111, USA along with a check for $10 (to cover printing and postage) payable to “Public Library of Science”. This article is digitally archived in PubMed Central and LOCKSS.

### DNA extraction, PCR amplification for direct sequencing and DNA sequencing conditions

DNA was extracted according to Inderbitzin et al. [27]. For extraction of DNA from the *V. dahliae* type material, the same protocol as for extractions from mycelium recovered from agar plates was used.

### Loci used for phylogenetic analyses and primer design

Five loci were used in this study, including *actin* (*ACT*), *elongation factor 1-alpha* (*EF*), *glyceraldehyde-3-phosphate dehydrogenase* (*GPD*), *tryptophan synthase* (*TS*) and the ribosomal internal transcribed spacer region ITS. Primers used to PCR amplify and sequence the ITS region were ITS1-F [81], ITS4 and ITS5 [82]. The *TS* region of *Verticillium albo-atrum* was at times PCR amplified and sequenced with primer pair VTs5f (5′-ACC TAT GTC ACT GCC GGC T-3′) and VTs4r (5′-CAA TGA AGC CGT TGA CGC C-3′). For more details on *TS* as well as the remaining loci, PCR conditions and DNA sequencing, see Inderbitzin et al. [27].

### *MAT* screening

Isolates in *V. alfalfae* and *V. nonalfalfae* were screened for presence of *MAT1-1* and *MAT1-2* idiomorphs according to Inderbitzin et al. [27].

### Phylogenetic analyses

Besides the single-locus *ACT*, *EF*, *GPD*, *TS* and ITS datasets, a combined, four-locus dataset comprised of concatenated *ACT*, *EF*, *GPD* and *TS* datasets was analyzed.

The datasets were analyzed as outlined in Inderbitzin et al. [27] using three different algorithms. DNA sequences were assembled and aligned in Geneious v4.8.5 [83]. Single-locus datasets were analyzed under the maximum parsimony criterion using PAUP v.4.0b 10 [84]. The combined four-locus dataset was analyzed using parsimony, maximum likelihood as implemented in PAUP v.4.0b 10, as well as MrBayes v3.0b4 [85] implementing a Bayesian approach to inferring phylogenies.

Most parsimonious trees were inferred using 30 random addition replicates. Otherwise, default settings were used, including treating insertion/deletion gaps as missing data. Bootstrap support values were based on 500 replicates. Maximum likelihood analyses were done using default settings and 30 random addition replicates, bootstrap supports were based on 415 replicates. Bayesian analyses were performed with default settings, running four chains over 10 million generations and sampling each 100^th^ tree. The first 1000 of the 10,000 saved trees were omitted and the consensus tree was based on the remaining 9,000 trees. Maximum likelihood and Bayesian analyses implemented an optimal model of DNA sequence evolution determined using Modeltest 3.7 [86]. All analyses were run with a single representative of each haplotype.

## Supporting Information

Figure S1 **Phylogenetic tree of** ***Verticillium*** **based on the ITS dataset comprising 74 taxa and 514 characters.** Shown is the single most parsimonious tree, 94 steps in length. Isolates are represented by a strain identifier; species are delimited by a vertical bar followed by a name. Branches with 100% bootstrap support are in bold, other support values above 70% are given by the branches.
(TIF)

Figure S2 **Phylogenetic tree of** ***Verticillium*** **based on the** ***ACT*** **dataset comprising 77 taxa and 638 characters.** Shown is one of the nine equally parsimonious trees, 427 steps in length. Isolates are represented by a strain identifier; species are delimited by a vertical bar followed by a name. Branches with 100% bootstrap support are in bold, other support values above 70% are given by the branches.
(TIF)

Figure S3 **Phylogenetic tree of** ***Verticillium*** **based on the** ***EF*** **dataset comprising 77 taxa and 614 characters.** Shown is one of the 12 equally parsimonious trees, 599 steps in length. Isolates are represented by a strain identifier; species are delimited by a vertical bar followed by a name. Branches with 100% bootstrap support are in bold, other support values above 70% are given by the branches.
(TIF)

Figure S4 **Phylogenetic tree of** ***Verticillium*** **based on the** ***GPD*** **dataset comprising 77 taxa and 781 characters.** Shown is one of the 2 equally parsimonious trees, 430 steps in length. Isolates are represented by a strain identifier; species are delimited by a vertical bar followed by a name. Branches with 100% bootstrap support are in bold, other support values above 70% are given by the branches.
(TIF)

Figure S5 **Phylogenetic tree of** ***Verticillium*** **based on the** ***TS*** **dataset comprising 77 taxa and 625 characters.** Shown is one of the 396 equally parsimonious trees, 565 steps in length. Isolates are represented by a strain identifier; species are delimited by a vertical bar followed by a name. Branches with 100% bootstrap support are in bold, other support values above 70% are given by the branches.
(TIF)

Table S1 (DOC)
