# Supplementary figures and images for: Phylogenetics and Taxonomy of the Fungal Vascular Wilt Pathogen Verticillium, with the Descriptions of Five New Species
Source: PLoS One. 2011 Dec 7;6(12):e28341. doi: 10.1371/journal.pone.0028341 (PMC3233568; doi:10.1371/journal.pone.0028341)

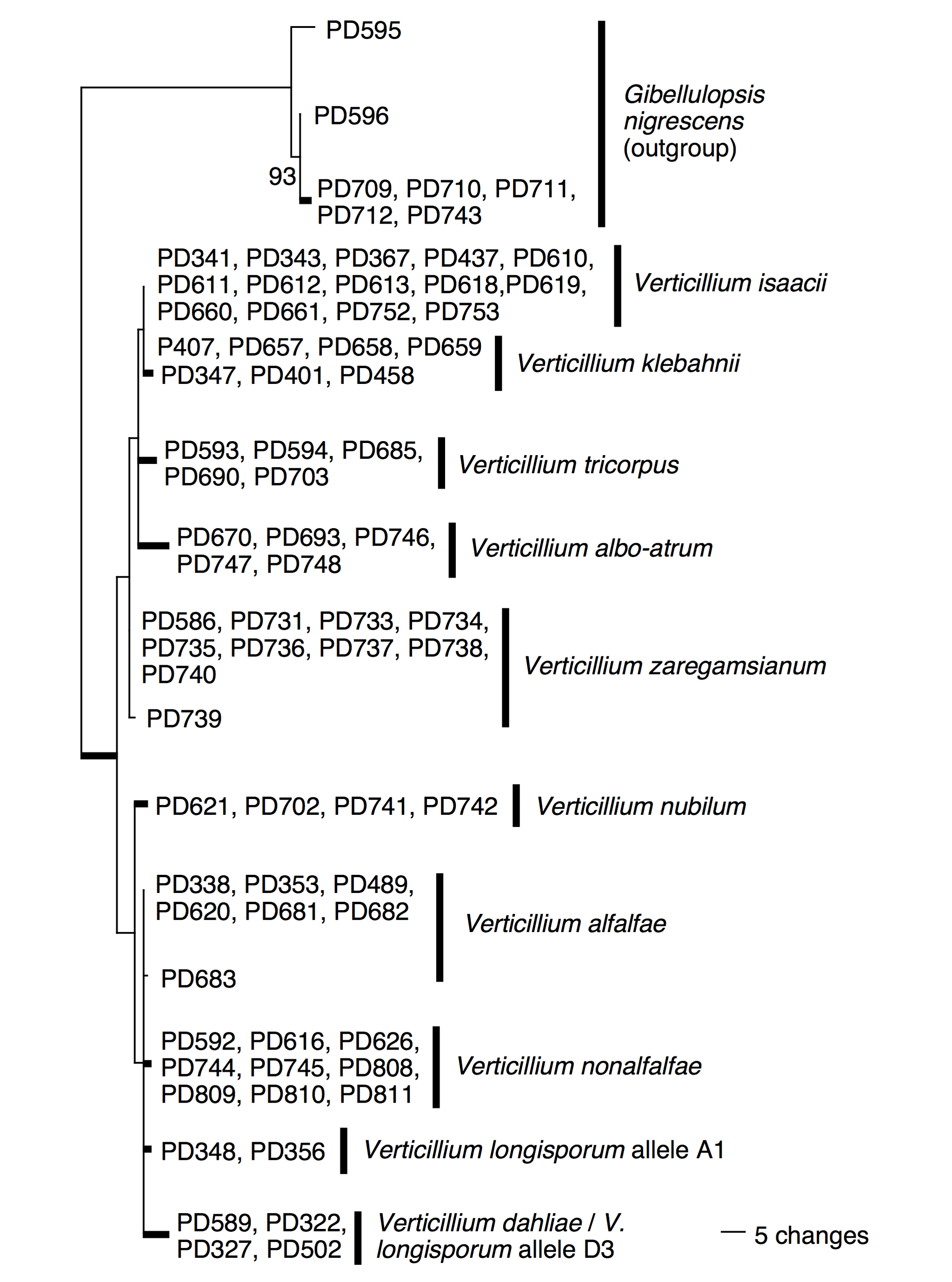

Supplement: Figure S1 — Phylogenetic tree of Verticillium based on the ITS dataset comprising 74 taxa and 514 characters. Shown is the single most parsimonious tree, 94 steps in length. Isolates are represented by a strain identifier; species are delimited by a vertical bar followed by a name. Branches with 100% bootstrap support are in bold, other support values above 70% are given by the branches. (TIF) [file pone.0028341.s001.tif]

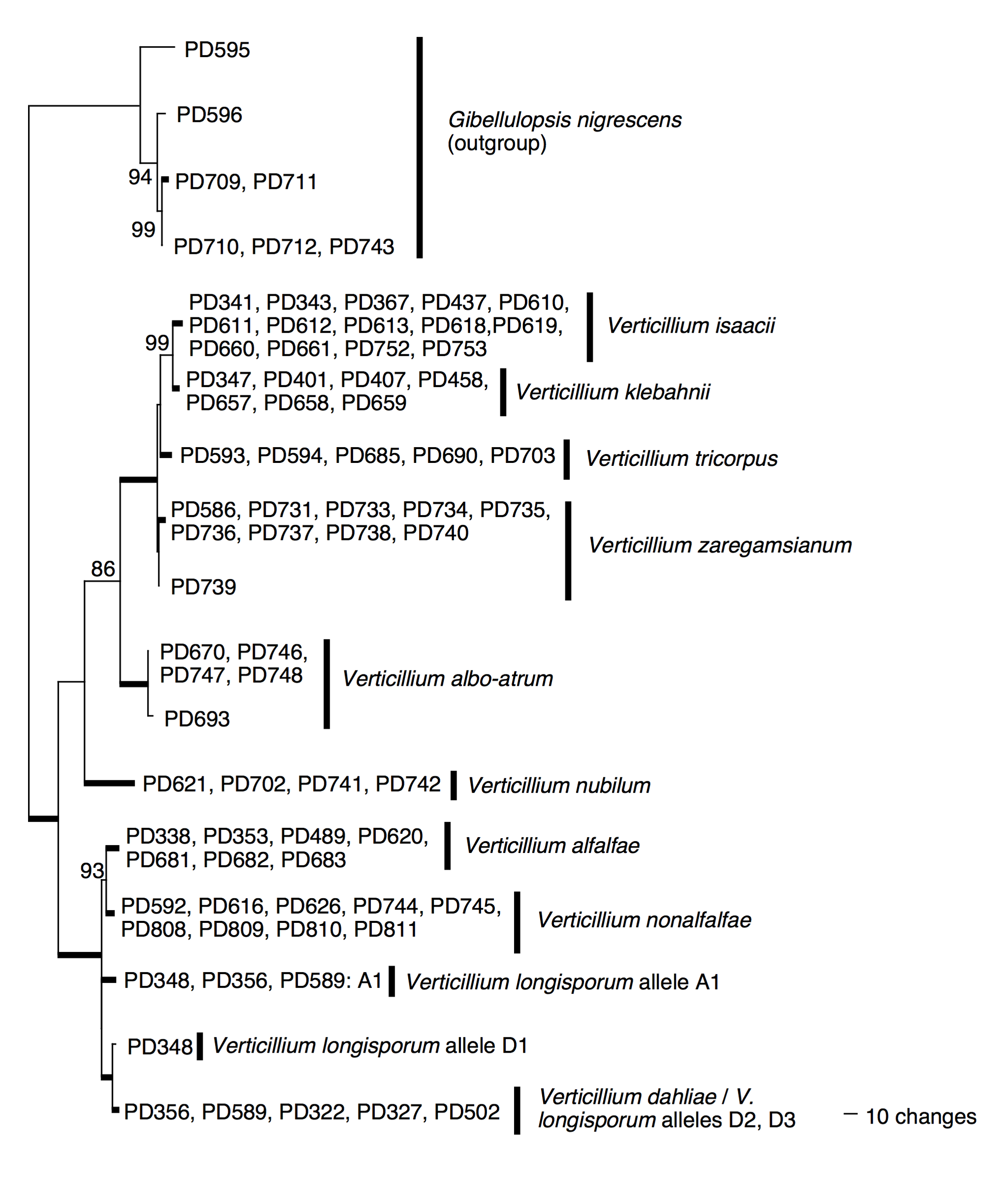

Supplement: Figure S2 — Phylogenetic tree of Verticillium based on the ACT dataset comprising 77 taxa and 638 characters. Shown is one of the nine equally parsimonious trees, 427 steps in length. Isolates are represented by a strain identifier; species are delimited by a vertical bar followed by a name. Branches with 100% bootstrap support are in bold, other support values above 70% are given by the branches. (TIF) [file pone.0028341.s002.tif]

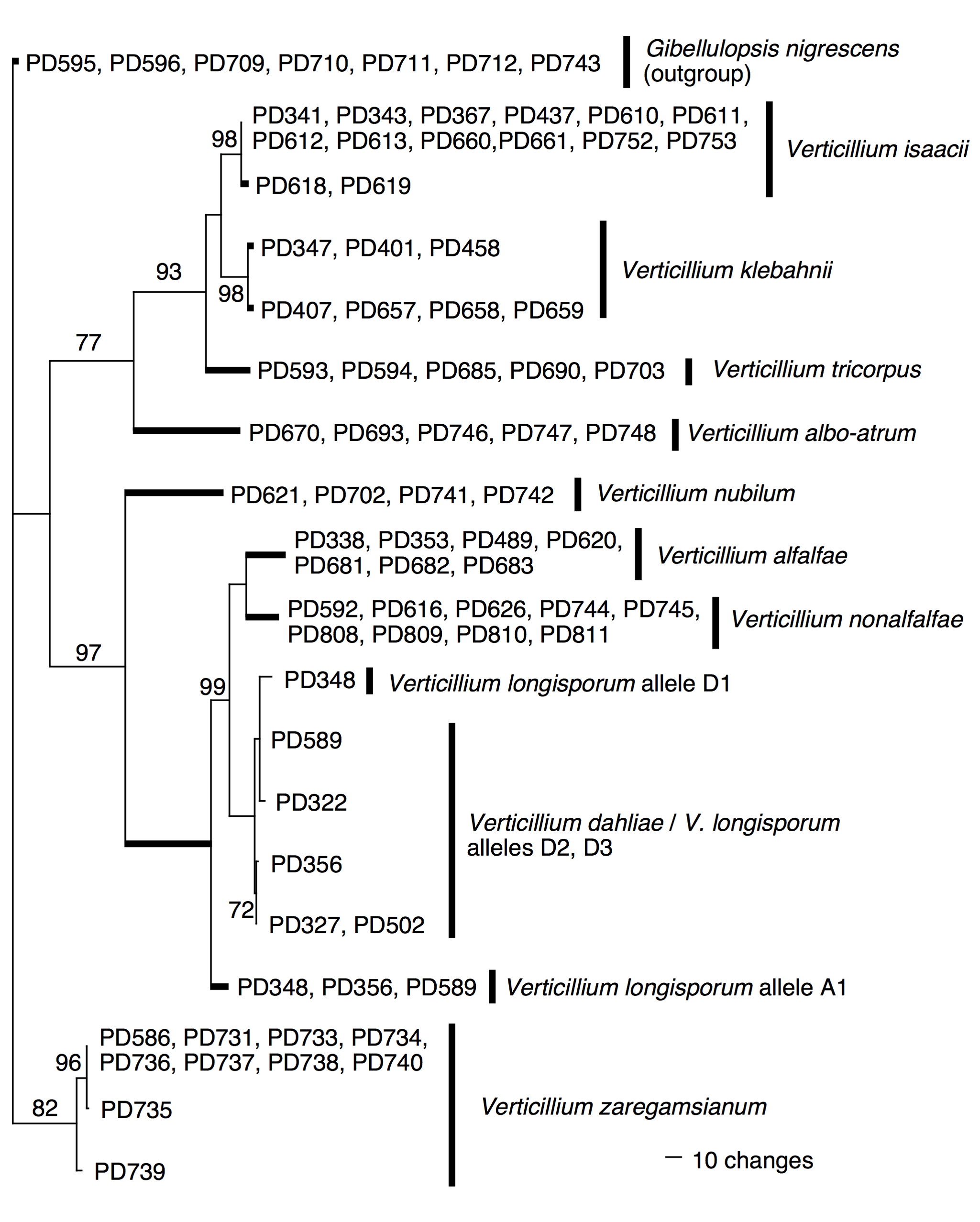

Supplement: Figure S3 — Phylogenetic tree of Verticillium based on the EF dataset comprising 77 taxa and 614 characters. Shown is one of the 12 equally parsimonious trees, 599 steps in length. Isolates are represented by a strain identifier; species are delimited by a vertical bar followed by a name. Branches with 100% bootstrap support are in bold, other support values above 70% are given by the branches. (TIF) [file pone.0028341.s003.tif]

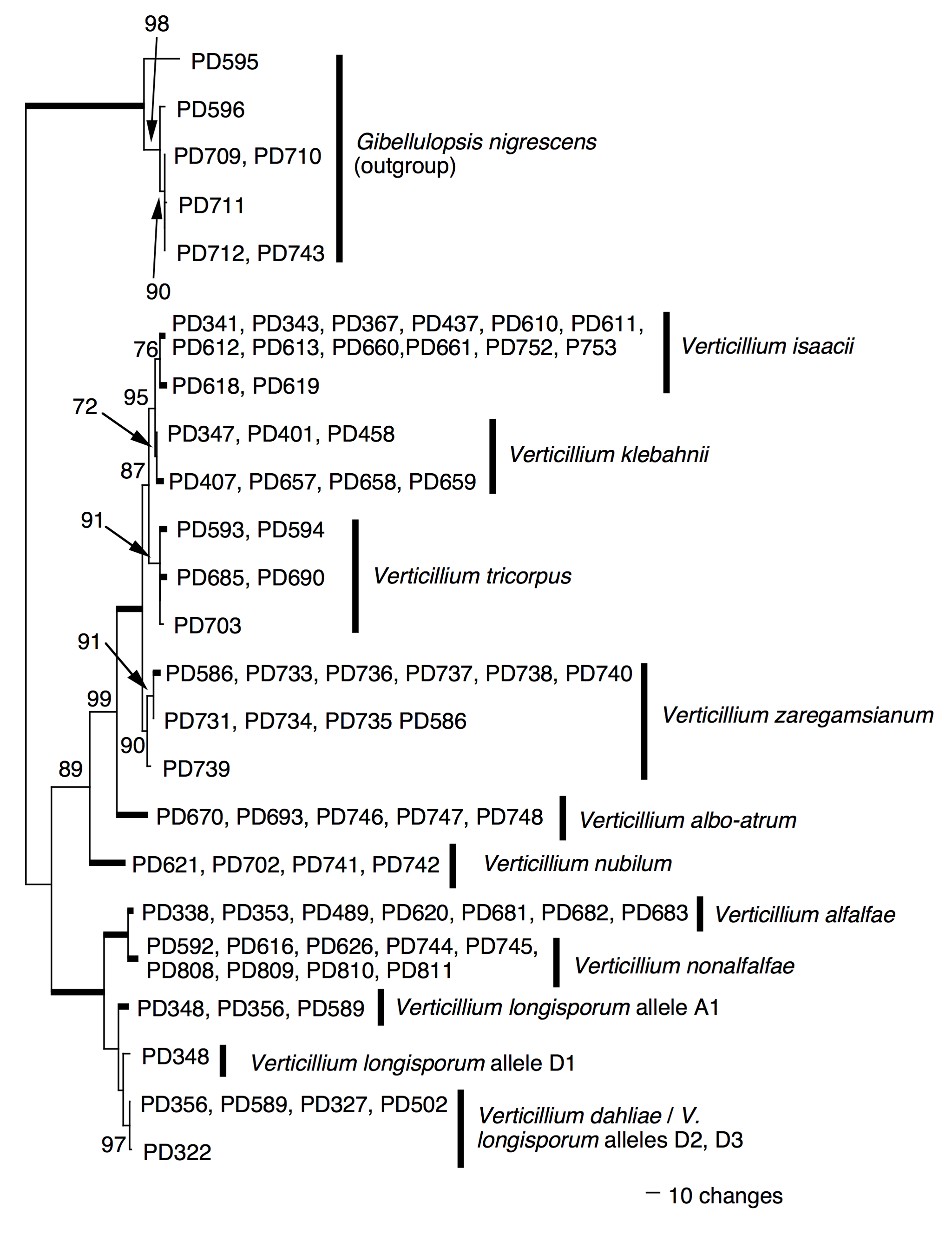

Supplement: Figure S4 — Phylogenetic tree of Verticillium based on the GPD dataset comprising 77 taxa and 781 characters. Shown is one of the 2 equally parsimonious trees, 430 steps in length. Isolates are represented by a strain identifier; species are delimited by a vertical bar followed by a name. Branches with 100% bootstrap support are in bold, other support values above 70% are given by the branches. (TIF) [file pone.0028341.s004.tif]

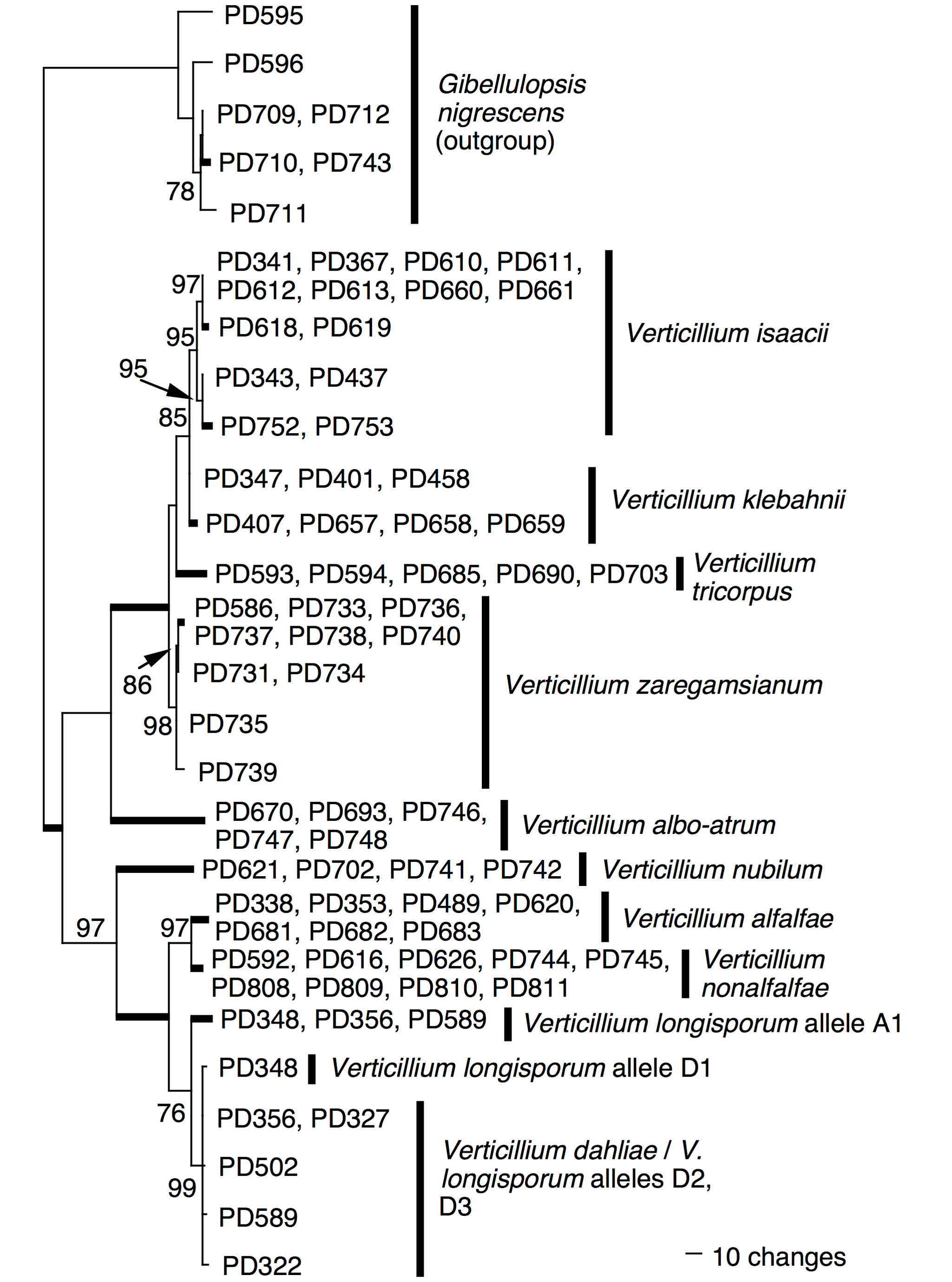

Supplement: Figure S5 — Phylogenetic tree of Verticillium based on the TS dataset comprising 77 taxa and 625 characters. Shown is one of the 396 equally parsimonious trees, 565 steps in length. Isolates are represented by a strain identifier; species are delimited by a vertical bar followed by a name. Branches with 100% bootstrap support are in bold, other support values above 70% are given by the branches. (TIF) [file pone.0028341.s005.tif]
