# Supplementary material for: Phylogenetics and Taxonomy of the Fungal Vascular Wilt Pathogen Verticillium, with the Descriptions of Five New Species
Source: PLoS One. 2011 Dec 7;6(12):e28341. doi: 10.1371/journal.pone.0028341 (PMC3233568; doi:10.1371/journal.pone.0028341)
Supplement: Table S1 — (DOC) [file pone.0028341.s006.doc]

Table S1. Fungal isolates used in this study. Given are our strain identifiers, additional strain identifiers, species names, host scientific and common names, locations, collection dates and strain origins.

| Strain identifiers used in this study | Other strain identifiers | Species^A^ | Host | Host common name^B^ | Location | Collection date | Source |
| --- | --- | --- | --- | --- | --- | --- | --- |
| PD322 | Ls.17, CBS 130341, NRRL 54785 | *Verticillium dahliae* Kleb. | *Lactuca sativa* L. | lettuce | Watsonville, CA, USA | 1996 | Subbarao Lab Collection |
| PD327 | Ca.59, B, T2 | *Verticillium dahliae* | *Capsicum annuum* L. | bell pepper^C^ | Gilroy CA, USA | 1996 | Subbarao Lab Collection |
| PD338 | Ms.107 | *Verticillium alfalfae* Inderb. et al. | *Medicago sativa* L. | alfalfa | Pennsylvania, USA | 1986 | Subbarao Lab Collection |
| PD341 | Ls.183 | *Verticillium* *isaacii* Inderb. et al. | *Lactuca sativa* | lettuce | California, USA | 1997-2001 | Subbarao Lab Collection |
| PD343 | Cs.234 | *Verticillium* *isaacii* | *Cynara scolymus* L. | globe artichoke | California, USA | 1999 | Subbarao Lab Collection |
| PD347 | Cs.456 | *Verticillium* *klebahnii* Inderb. et al. | *Cynara scolymus* | globe artichoke | California, USA | 1999 | Subbarao Lab Collection |
| PD348 | Bob.70, 90-02 | *Verticillium* *longisporum* (C. Stark) Karapapa, Bainbr. & Heale lineage A1/D1 | *Brassica oleracea* var*. botrytis* L. | cauliflower^C^ | Salinas, CA, USA | 1990 | Subbarao Lab Collection |
| PD353 | Ms.102, VP-1 | *Verticillium alfalfae* | *Medicago sativa* | alfalfa | Smullton, PA, USA | 1986 | B. W. Pennypacker |
| PD356 | Ar.139 | *Verticillium* *longisporum* lineage A1/D2 | *Armoracia rusticana* P.G. Gaertn., B. Mey. & Scherb. | horseradish | Illinois, USA | 1997 | Subbarao Lab Collection |
| PD367 | Ss.454 | *Verticillium* *isaacii* | *Solanum physalifolium* Rusby | hairy nightshade | Salinas, CA, USA | 2001 | Subbarao Lab Collection |
| PD401 | Ls.14, CBS 130344, NRRL 54789 | *Verticillium* *klebahnii* | *Lactuca sativa* | lettuce | Watsonville, CA, USA | 1996 | Subbarao Lab Collection |
| PD407 | Ls.911 | *Verticillium* *klebahnii* | *Lactuca sativa* | lettuce | Watsonville, CA, USA | 2006 | Subbarao Lab Collection |
| PD437 | Ls.838 | *Verticillium* *isaacii* | *Lactuca sativa* | lettuce | Watsonville, CA, USA | 2005 | Subbarao Lab Collection |
| PD458 | Ls.644 | *Verticillium* *klebahnii* | *Lactuca sativa* | lettuce | Watsonville, CA, USA | 2004 | Subbarao Lab Collection |
| PD489 | L-715, CBS 130603, NRRL 54790 | *Verticillium alfalfae* | *Medicago sativa* | NA | USA | ? | Tom Gordon |
| PD502 | Acer.875 | *Verticillium dahliae* | *Acer* sp. | maple | Greenfield, WI, USA | 2006 | Glen Stanosz |
| PD586 | St4 | *Verticillium* *zaregamsianum* Inderb. et al. | *Matthiola incana* (L.) Ait. f. | tenweeks stock | Chiba, Japan | 2000 | Toshiyuki Usami |
| PD592 | HP, CBS 130339, NRRL 54791 | *Verticillium* *nonalfalfae* Inderb. et al. | *Solanum tuberosum* L. | Irish potato | Hokkaidou, Japan | ? | Toshiyuki Usami |
| PD593 | CE98Vt1 | *Verticillium* *tricorpus* I. Isaac | *Solanum tuberosum* | Irish potato | Japan | 1998 | Toshiyuki Usami |
| PD594 | CE20VtLe3 | *Verticillium* *tricorpus* | *Solanum lycopersicum* var*. lycopersicum* L. | garden tomato | Chiba, Japan | 2000 | Toshiyuki Usami |
| PD595 | CE20VnAc1 | *Gibellulopsis* *nigrescens* (Pethybr.) Zare, W. Gams & Summerb. | *Anemone coronaria* L. | anemone | Chiba, Japan | 2000 | Toshiyuki Usami |
| PD596 | CE98VnSm1 | *Gibellulopsis* *nigrescens* | *Solanum melongena* L*.* | eggplant | Chiba, Japan | 1998 | Toshiyuki Usami |
| PD610 | Ls.432 | *Verticillium* *isaacii* | *Lactuca sativa* | lettuce | California, USA | 1997-2001 | Subbarao Lab Collection |
| PD611 | Ls.441 | *Verticillium* *isaacii* | *Lactuca sativa* | lettuce | California, USA | 1997-2001 | Subbarao Lab Collection |
| PD612 | Ls.442 | *Verticillium* *isaacii* | *Lactuca sativa* | lettuce | California, USA | 1997-2001 | Subbarao Lab Collection |
| PD613 | Ls.443 | *Verticillium* *isaacii* | *Lactuca sativa* | lettuce | California, USA | 1997-2001 | Subbarao Lab Collection |
| PD616 | 1953 | *Verticillium* *nonalfalfae* | *Humulus lupulus* L. | common hop | UK | 1972 | Dez Barbara |
| PD618 | 1988 | *Verticillium* *isaacii* | *Solanum lycopersicum* var*. lycopersicum* | garden tomato | UK | 1979 | Dez Barbara |
| PD619 | 151 | *Verticillium* *isaacii* | soil | NA | Canada | 1989 | Dez Barbara |
| PD620 | STR1 | *Verticillium alfalfae* | *Medicago sativa* | alfalfa | Canada | ? | Dez Barbara |
| PD621 | IMI 130213 | *Verticillium* *nubilum* Pethybr. | Mushroom compost | NA | UK | ? | Dez Barbara |
| PD626 | 1974 | *Verticillium* *nonalfalfae* | *Humulus lupulus* | common hop | UK | 1980 | Dez Barbara |
| PD657 | 730 a2 | *Verticillium* *klebahnii* | *Lactuca sativa* | lettuce | Washington, USA | 2009 | Subbarao Lab Collection |
| PD658 | 730 a9 | *Verticillium* *klebahnii* | *Lactuca sativa* | lettuce | Washington, USA | 2009 | Subbarao Lab Collection |
| PD659 | 730 C2 | *Verticillium* *klebahnii* | *Lactuca sativa* | lettuce | Washington, USA | 2009 | Subbarao Lab Collection |
| PD660 | 730 C6, CBS 130343, NRRL 54792 | *Verticillium* *isaacii* | *Lactuca sativa* | lettuce | California, USA | ? | Subbarao Lab Collection |
| PD661 | 730 C8 | *Verticillium* *isaacii* | *Lactuca sativa* | lettuce | Washington, USA | 2009 | Subbarao Lab Collection |
| PD670 | V.10, IPP 0323 | *Verticillium albo-atrum* Reinke & Berthold | *Solanum tuberosum* | Irish potato | Wisconsin, USA | ? | Andreas von Tiedemann |
| PD681 | 220 | *Verticillium alfalfae* | *Medicago sativa* | alfalfa | USA | 1982 | Milton Typas |
| PD682 | MAFF 235137 | *Verticillium alfalfae* | *Medicago sativa* | alfalfa | Hokkaidou, Japan | 1981 | National Institute of Agrobiological Sciences, Japan (NIAS) |
| PD683 | MAFF 235138 | *Verticillium alfalfae* | *Medicago sativa* | alfalfa | Hokkaidou, Japan | 1980 | National Institute of Agrobiological Sciences, Japan (NIAS) |
| PD685 | MAFF 712235 | *Verticillium* *tricorpus* | *Delphinium* sp. | larkspur | Japan | 1999 | National Institute of Agrobiological Sciences, Japan (NIAS) |
| PD687 | CBS 124.64, MUCL 9802, NRRL 54793 | *Verticillium* *longisporum* lineage A1/D3 | *Armoracia rusticana* | horseradish | Niedersachsen, Germany | 1959 | Centraalbureau voor Schimmelcultures (CBS) |
| PD690 | CBS 447.54, NRRL 54794 | *Verticillium* *tricorpus* | *Solanum lycopersicum* var*. lycopersicum* | garden tomato | UK | 1950 | Centraalbureau voor Schimmelcultures (CBS) |
| PD693 | IRAN 500 C, IMI 276673, CBS 101242 | *Verticillium albo-atrum* | *Solanum tuberosum* | Irish potato | UK | 1983 | Rasoul Zare |
| PD702 | IRAN 546 C, 37311, IMI 278734 | *Verticillium* *nubilum* | *Solanum tuberosum* | Irish potato | UK | 1983 | Rasoul Zare |
| PD703 | IRAN 490 C, IMI 335344 | *Verticillium* *tricorpus* | *Dianthus caryophyllus* L. | carnation | Netherlands | 1989 | Rasoul Zare |
| PD709 | 23.2 | *Gibellulopsis* *nigrescens* | *Papaver somniferum* L. | opium poppy | Poltavska, oblast, Ustimovka, Ukraine | 2003 | Elena L. Gasich, Philipp Gannibal |
| PD710 | 23.3 | *Gibellulopsis* *nigrescens* | *Papaver somniferum* | opium poppy | Poltavska, oblast, Ustimovka, Ukraine | 2003 | Elena L. Gasich, Philipp Gannibal |
| PD711 | 18.5 | *Gibellulopsis* *nigrescens* | *Papaver somniferum* | opium poppy | Poltavska, oblast, Ustimovka, Ukraine | 2003 | Elena L. Gasich, Philipp Gannibal |
| PD712 | 18.11 | *Gibellulopsis* *nigrescens* | *Papaver somniferum* | opium poppy | Poltavska, oblast, Ustimovka, Ukraine | 2003 | Elena L. Gasich, Philipp Gannibal |
| PD731 | MIH001 | *Verticillium* *zaregamsianum* | *Lactuca sativa* | lettuce | Hyogo, Japan | 2002 | Toshiyuki Usami, Takeshi Kanto |
| PD733 | Shichi6 | *Verticillium* *zaregamsianum* | *Lactuca sativa* | lettuce | Hyogo, Japan | 2007 | Toshiyuki Usami, Takeshi Kanto |
| PD734 | Shichi12 | *Verticillium* *zaregamsianum* | *Lactuca sativa* | lettuce | Hyogo, Japan | 2007 | Toshiyuki Usami, Takeshi Kanto |
| PD735 | KNty-2-2 | *Verticillium* *zaregamsianum* | *Lactuca sativa* | lettuce | Kagawa, Japan | 2005 | Toshiyuki Usami, Gan Kisaki |
| PD736 | eLTS-1, CBS 130342, NRRL 54795 | *Verticillium* *zaregamsianum* | *Lactuca sativa* | lettuce | Chiba, Japan | 2000 | Toshiyuki Usami, Yoshiyuki Ebihara |
| PD737 | eLTS-2 | *Verticillium* *zaregamsianum* | *Lactuca sativa* | lettuce | Chiba, Japan | 2000 | Toshiyuki Usami, Yoshiyuki Ebihara |
| PD738 | CE99Vt2 | *Verticillium* *zaregamsianum* | *Lactuca sativa* | lettuce | Chiba, Japan | 1999 | Toshiyuki Usami, Yoshiyuki Ebihara |
| PD739 | CE98Vt8 | *Verticillium* *zaregamsianum* | *Matthiola incana* | tenweeks stock | Chiba, Japan | 1998 | Toshiyuki Usami, Yoshiyuki Ebihara |
| PD740 | CE99Vt6 | *Verticillium* *zaregamsianum* | *Matthiola incana* | tenweeks stock | Chiba, Japan | 1999 | Toshiyuki Usami, Yoshiyuki Ebihara |
| PD741 | CBS 578.50 | *Verticillium* *nubilum* | soil | NA | East Malling Research, Station, UK | 1949 | Centraalbureau voor Schimmelcultures (CBS) |
| PD742 | CBS 457.51, NRRL 54796 | *Verticillium* *nubilum* | soil | NA | UK | 1951 | Centraalbureau voor Schimmelcultures (CBS) |
| PD743 | 5009 | *Gibellulopsis* *nigrescens* | *Solanum tuberosum* | Irish potato | Prince Edward Island, Canada | 2009 | Bud Platt |
| PD744 | 1852 | *Verticillium* *nonalfalfae* | potato soil | NA | Cuba | 1985 | Bud Platt |
| PD745 | 1856 | *Verticillium* *nonalfalfae* | *Spinacia oleracea* L. | spinach^D^ | Manitoba, Canada | 1985 | Bud Platt |
| PD746 | 4901 | *Verticillium albo-atrum* | potato soil | NA | New Brunswick, Canada | 1990 | Bud Platt |
| PD747 | V104, CBS 130340, NRRL 54797 | *Verticillium albo-atrum* | potato soil | NA | Prince Edward Island, Canada | 1989 | Bud Platt |
| PD748 | V112 | *Verticillium albo-atrum* | potato soil | NA | Prince Edward Island, Canada | 1989 | Bud Platt |
| PD752 | #3(5) | *Verticillium* *isaacii* | *Spinacia oleracea* | spinach^D^ | Washington, USA | 2009 | Subbarao Lab Collection |
| PD753 | #3(25) | *Verticillium* *isaacii* | *Spinacia oleracea* | spinach^D^ | Washington, USA | 2009 | Subbarao Lab Collection |
| PD808 | Pec | *Verticillium* *nonalfalfae* | *Humulus lupulus* | common hop | Slovenia | 2000 | Sebastjan Radišek |
| PD809 | Mo3 | *Verticillium* *nonalfalfae* | *Humulus lupulus* | common hop | Slovenia | 1999 | Sebastjan Radišek |
| PD810 | Surf | *Verticillium* *nonalfalfae* | *Petunia* sp. | petunia | Slovenia | 2000 | Sebastjan Radišek |
| PD811 | Or99 | *Verticillium* *nonalfalfae* | *Humulus lupulus* | common hop | Slovenia | 1999 | Sebastjan Radišek |

^A^Lineage affiliations are given for *V. longisporum*.

^B^The common names follow www.ITIS.gov accessed on June 6, 2010, except indicated otherwise.

^C^Common name differing from www.ITIS.gov accessed on June 6, 2010

^D^Isolated from seed.
